# Nuclear Localization of Suppressor of Cytokine Signaling-1 Regulates Local Immunity in the Lung

**DOI:** 10.3389/fimmu.2016.00514

**Published:** 2016-11-18

**Authors:** Jana Zimmer, Michael Weitnauer, Sébastien Boutin, Günter Küblbeck, Sabrina Thiele, Patrick Walker, Felix Lasitschka, Lars Lunding, Zane Orinska, Christina Vock, Bernd Arnold, Michael Wegmann, Alexander Dalpke

**Affiliations:** ^1^Department of Infectious Diseases, Medical Microbiology and Hygiene, University Hospital Heidelberg, Heidelberg, Germany; ^2^Translational Lung Research Center Heidelberg (TLRC), Heidelberg, Germany; ^3^German Center for Lung Research (DZL), Germany; ^4^German Cancer Research Center (DKFZ), Heidelberg, Germany; ^5^Institute of Pathology, University Hospital Heidelberg, Heidelberg, Germany; ^6^Division of Asthma Mouse Model, Research Center Borstel, Borstel, Germany; ^7^Airway Research Center North, Borstel, Germany; ^8^Division of Experimental Pneumology, Prority Area Asthma & Allergy, Research Center Borstel, Borstel, Germany

**Keywords:** rodent, inflammation, signal transduction, transgenic mice, lung, SOCS1

## Abstract

Suppressor of cytokine signaling 1 (SOCS1) is a negative feedback inhibitor of cytoplasmic Janus kinase and signal transducer and activator of transcription (STAT) signaling. SOCS1 also contains a nuclear localization sequence (NLS), yet, the *in vivo* importance of nuclear translocation is unknown. We generated transgenic mice containing mutated Socs1ΔNLS that fails to translocate in the cell nucleus (*MGL*^*tg*^ mice). Whereas mice fully deficient for SOCS1 die within the first 3 weeks due to excessive interferon signaling and multiorgan inflammation, mice expressing only non-nuclear *Socs1ΔNLS* (*Socs1^−/−^MGL^tg^* mice) were rescued from early lethality. Canonical interferon gamma signaling was still functional in *Socs1^−/−^MGL^tg^* mice as shown by unaltered tyrosine phosphorylation of STAT1 and whole genome expression analysis. However, a subset of NFκB inducible genes was dysregulated. *Socs1^−/−^MGL^tg^* mice spontaneously developed low-grade inflammation in the lung and had elevated Th2-type cytokines. Upon ovalbumin sensitization and challenge, airway eosinophilia was increased in *Socs1^−/−^MGL^tg^* mice. Decreased transepithelial electrical resistance in trachea epithelial cells from *Socs1^−/−^MGL^tg^* mice suggests disrupted epithelial cell barrier. The results indicate that nuclear SOCS1 is a regulator of local immunity in the lung and unravel a so far unrecognized function for SOCS1 in the cell nucleus.

## Introduction

The suppressor of cytokine signaling (SOCS) family is important for negative feedback inhibition of Janus kinases (JAKs) and signal transducer and activator of transcription (STAT) signaling. All eight members, namely SOCS1–7 and CIS, share key structural elements such as the central cytokine-inducible Src-homology 2 (SH2) domain and the shared C-terminal SOCS-box (1, 2). Both SOCS1 and SOCS3 additionally contain a kinase inhibitory region (KIR) by which they can act as a pseudosubstrate for JAKs (3). SOCS1 was first described in 1997 as a negative feedback inhibitor of cytoplasmic JAK/STAT signaling (4–6). By the means of the extended SH2 subdomain (eSS) and the KIR, SOCS1 directly binds to JAK2, thereby inhibiting its catalytic activity (3). SOCS1 has further been shown to occupy binding sites for STATs by interacting with interferon receptor domains (7, 8). Finally, the SOCS box in SOCS1 mediates interactions with elongins B and C and acts as an E3 ubiquitin ligase that targets JAKs or cytokine receptor complexes for proteasomal degradation (9, 10). In addition to JAK/STAT signaling, SOCS1 has been shown to act as a cross talk inhibitor for TLR signaling pathways (11, 12). Besides indirect paracrine inhibition of TLR signaling by IFNβ (13), SOCS1 additionally contributes to direct negative regulation by interacting with components of the TLR signaling pathway (12, 14).

Canonical IFNγ signaling functions by binding to the IFNγ receptor complex, activating Janus kinases (JAK1/2), and subsequent phosphorylation of STAT1 that dimerizes and translocates into the nucleus. Once in the nucleus, activated pY-STAT1 binds to gamma-activated sequence (GAS) elements within the promoters of IFNγ-responsive genes (ISGs) – mostly, “canonical” antiviral genes (15, 16). While pY-STAT1 is thought to be pivotal for the IFNγ response, a number of studies have shown that pY-STAT1-independent pathways also exist (17–19). There is emerging evidence that STAT-independent pathways play important roles in mediating signals for the generation of IFNγ-responses such as the mitogen-activated protein kinase (MAPK) or PI3K/AKT pathway (20). It has been previously shown that IFNAR1, TYK2, and STAT1 may translocate into the nucleus (21, 22). The presence of unphosphorylated STAT1 in the cell nucleus has been shown to increase expression of only a subset of “non-canonical” IFNγ-induced genes that are pY-STAT1-independent (23).

*Socs1^−/−^* mice die within 2–3 weeks due to unlimited IFNγ signaling leading to multiorgan inflammation (24–26). Deletion of the SOCS box of SOCS1 delays the onset of the disease (27). Alleviation from the lethal phenotype of *Socs1^−/−^* mice can be achieved by backcrossing to IFNγ^−/−^mice; however, these mice develop polycystic kidneys as well as chronic inflammation (28). Moreover, *Socs1^−/−^* mice can be rescued by backcrossing to either *STAT4^−/−^*, or *STAT6^−/−^* mice (25, 29), or *Rag^−/−^* mice (30), revealing an important role of SOCS1 in T cells. Since *Socs1^−/−^* mice have defective thymocyte development, and overexpression of *Socs1* impairs pre-TCR-induced thymocyte proliferation, inhibition of cytokine signaling has important influence on T cell differentiation (31, 32).

In 2008, a nuclear localization sequence (NLS) has been identified in SOCS1 located between the central SH2 domain and the SOCS box (amino acids 159–173). The NLS resulted in translocation of the protein into the cell nucleus (33, 34). Substitution of this sequence with the respective region of SOCS3 showed loss of nuclear localization, whereas fusion of the SOCS1–NLS to the cytoplasmic SOCS family member CIS induced nuclear localization (33). It has been shown that SOCS1 directly interacts with the tumor suppressor p53 leading to activation of p53 *via* phosphorylation (35). Moreover, SOCS1 induces proteasomal degradation of NFκB (36, 37) and, in particular, it interacts with the NFκB subunit p65 in the cell nucleus, thereby limiting induction of a subset of NFκB dependent genes (38).

However, the function of SOCS1 in the cell nucleus *in vivo* remains elusive. Therefore, we generated a transgenic mouse that only expresses a non-nuclear mutant SOCS1. Mice with transgenic expression of a bacterial artificial chromosome (BAC) containing a mutated *Socs1* locus with non-nuclear *Socs1ΔNLS*, e*GFP*, and *LuciferaseCBG99* (*MGL*) were generated and backcrossed to *Socs1^−/−^* mice. *Socs1^−/−^MGL^tg^* mice survived the early lethal phenotype of *Socs1^−/−^* mice, showed unaltered canonical IFNγ-signaling, yet, displayed signs of low-grade airway inflammation and Th2 deviation. Decreased transepithelial electrical resistance (TER) in trachea epithelial cells from *Socs1^−/−^MGL^tg^* mice suggests disrupted epithelial integrity. *Socs1^−/−^MGL^tg^* mice present a valuable tool to study the nuclear function of SOCS1 *in vivo* and allow investigating local immune regulation in the lung by nuclear SOCS1.

## Materials and Methods

### Mice

C57BL/6 mice were purchased from Charles River Laboratories. Breeding occurred under specific pathogen-free conditions in the animal facility (IBF, Heidelberg, Germany). Socs1^+/−^ mice (C57/Bl6.129Sv-Socs1tmWsa/Uhg) were first described by Starr et al. (26). MGL-transgenic mice were generated by pronucleus injection using a BAC containing a part of chromosome #16 (10.78–10.80 Mb) including a mutated *Socs1* locus with non-nuclear *Socs1ΔNLS, eGFP* (codon optimized for mouse and human), and *LuciferaseCBG99* (Click Beetle Green from Pyrophorus plagiophalam), termed MGL (RP23-360O7). Pronucleus injection resulted in 12 transgenic founder mice, C57Bl6-tg(Socs1-MGL)Uhg. This work was done by Prof. Dr. Bernd Arnold and Günter Küblbeck (DKFZ, Heidelberg, Germany) in cooperation with Frank Zimmermann (IBF) and Patrick Walker. Mice are genotyped at an age of 2 weeks using PCR detecting *Socs1 wild-type (wt), Socs1 knockout, Socs1 MGL*, and *β2microglobulin* (*β2M*) (primer sequences, see Table S1 in Supplementary Material). Breeding, sacrificing, and dissection were approved and experiments properly recorded and reported to the regional commission in Karlsruhe (permit number 35-9185.81/G-54/14).

### Reagents

RPMI 1640 was purchased from Biochrom (Berlin, Germany). FCS was from Life Technologies (Carlsbad, CA, USA) and Penicillin and streptomycin were from PAA Laboratories (Pasching, Austria). PBS was obtained from PAN-Biotech (Aidenbach, Germany). IFNγ was purchased from Peprotech (#315-05, Hamburg, Germany), polyinosinic–polycytidylic acid (pI:C) from InvivoGen (Toulouse, France) and phosphorothioate-modified CpG-oligonucleotide 1668 from TIB Molbiol (Berlin, Germany). LPS from *Salmonella minnesota* was kindly provided by U. Seydel (Division of Biophysics, Research Center Borstel, Borstel, Germany).

### Cell Culture, Transfection, and Stimulation

RAW264.7 or NIH cells were cultured at 37°C and 5% CO_2_ in RPMI or DMEM, respectively. Cell culture medium was further supplemented with 10% (v/v) heat-inactivated fetal calf serum (FCS), penicillin (50 units/ml), and streptomycin (50 μg/ml) (P/S). For transfection of RAW264.7 or NIH cells, the transfection reagents JetPRIME (Polyplus, Illkirch, France) or PeqFect (peqlab Biotechnology, Erlangen, Germany) were used and transfection was performed according to the manufacturer’s protocol. Bone marrow-derived macrophages (BMM) were isolated from mice as described previously (39). Briefly, bone marrow cells were seeded into a 14.5 cm dish in DMEM plus FCS and P/S and differentiated using 30% (v/v) L929 supernatant (containing M-CSF) for 7 days. For cycloheximide (CHX) chase, 1 × 10^6^ BMMs were stimulated with IFNγ for 6 h and chased with 100 μg/ml CHX (Merck Millipore, MA, USA).

### Immunofluorescence Microscopy

NIH cells were grown on μ-slides (8-well, ibidi, Martinsried, Germany) and transfected with 0.5 μg *GFP-Socs1* or *GFP-Socs1ΔNLS* using PeqFect (peqlab Biotechnology, Erlangen, Germany). Where indicated, cells were stained with Hoechst (1 μg/ml) for 2 min or with CellMask™ Plasma Membrane Stain (ThermoFisher Scientific, Waltham, MA, USA, 1:1000) for 10 min at room temperature. Coverslips were mounted and analyzed by microscopy using a Leica TCS SP5 confocal microscope (Leica Microsystems, Wetzlar, Germany) equipped with a 488- and 561-nm laser, spectrophotometer prism, tunable detectors, and a HCX PL APO 63×/1.4 oil objective. All channels were recorded in a sequential order to avoid emission cross talk. A *z*-stack was recorded and presented as an overlay using ImageJ (National Institutes of Health). For quantification of the fluorescence, a region of interest (ROI) was set around the nucleus or the cytoplasm of a cell and intensity of the fluorescence was examined in a *z*-stack using ImageJ.

### Quantitative RT-PCR

Total RNA from 2.5 × 10^5^ cells was isolated using the peqGOLD RNA Kit (peqlab Biotechnology, Erlangen, Germany) according to the manufacturer’s protocol. After reverse transcription into cDNA using the High Capacity cDNA Reverse Transcription Kit (Applied Biosystems, Foster City, CA, USA), 5 μl cDNA (diluted 1:10) was used as template in a quantitative real-time PCR using SYBR Green FAST Mix (Applied Biosystems). Amplification and measurement was done in a StepOne Plus RT-PCR cycler (Applied Biosystems) in a 96-well format. Specificity of qPCR was controlled by non-template as well as no-RT samples and analysis of melting curves. Results are shown relative to the housekeeping gene *β-Actin (ActB)*. Primer sequences are given in Table S1 in Supplementary Material. qPCR for *Socs1 wt* was performed with TaqMan Fast Universal PCR Master Mix (Applied Biosystems) in combination with a forward primer (P1) binding in the SH2 domain and a reverse primer (P2) binding in the SOCS box and a FAM-labeled probe binding in the NLS of *Socs1*. SYBR green dye in combination with P1 and a reverse primer (P3) within the modified NLS region were used for the detection of *Socs1ΔNLS*. For detection of *total Socs1* (both *Socs1* and *Socs1ΔNLS*), the primers P1 and P2 were used. This qPCR strategy allows for specific detection of *Socs1, Socs1ΔNLS*, or *total Socs1*. The amplification efficiencies for both *Socs1 wt* (1.68) and *Socs1ΔNLS* (1.88) were adjusted for differences.

### Western Blotting

Also, 1 × 10^6^ BMMs or RAW264.7 cells were stimulated as indicated, subsequently washed with PBS, and lysed in Laemmli buffer [400 mM Tris–HCl, pH 6.8, 20% (v/v) β-mercaptoethanol, 40% (v/v) glycerol, 8% (w/v) SDS, and 0.4% (v/v) bromophenol blue]. After incubation for 10 min at 98°C, equal amounts of lysates were fractionated by 10% polyacrylamide gel (SDS-PAGE) and electrotransferred to Nitrocellulose membranes by a semidry blotting procedure [buffer: 25 mM Tris, 192 mM Glycin, 10% (v/v) methanol; 2.5 mA/cm^2^ for 1 h 15 min]. Blocking of unspecific binding was performed using 5% BSA solution in 1× TBST [1× TBS, 0.05% (v/v) Tween-20] for at least 1 h. Membranes were stained with antibodies against pY-STAT1 (Tyr701, #9167), STAT1 (#9172), IκBα (#9242), β-Actin (#4970) (Cell Signaling, Leiden, Netherlands; 1:1000), or hybridoma cell supernatant for SOCS1 detection (hybridoma cells newly generated by immunizing mice against the peptide RRITRASALLDA, Abmart, Shanghai, 1:20 dilution) overnight at 4°C. After three 10 min washing steps in 1× TBST at room temperature, blots were incubated with secondary antibodies for 1 h at RT [HRP-linked anti-mouse or anti-rabbit (Cell Signaling, Leiden, Netherlands)], followed by additional three 10 min washing steps in 1× TBST at room temperature. Proteins were detected using an enhanced chemiluminescence system (Western lightning™ plus ECL, Perkin-Elmer, Rodgau, Germany). Gels were imaged digitally, and contrast adjustments were applied to all parts of a figure. The prestained molecular weight marker was imaged separately (using transmitted light) and aligned to the digital images of the blots. The ladder is represented on the blots as black bars. Where indicated, membranes were stripped and reprobed. Densitometry was performed using ImageJ software (National Institutes of Health).

### Immunohistochemistry

For immunohistochemistry, lungs were fixed overnight in 4% (v/v) formalin and embedded in paraffin. Two micrometers lung sections were cut and stained for SOCS1 using the DAB staining method (Abcam, Cambridge, UK or Dako, Glostrup, Denmark). After deparaffination, demasking of the antibody followed using either citrate buffer [10 mM sodium citrate, 0.05% (v/v) Tween 20, pH 6.0] or EDTA buffer [1 mM EDTA, pH 8.0] for 15–45 min in a steamer. Incubation with peroxidase-blocking solution and protein blocking solution was followed by incubation with the anti-SOCS1 antibody at varying concentrations (1:50–1:2000) at 4°C overnight. The commercially available antibodies from cell signaling (#3950, Leiden, Netherlands), Abcam (#ab-9870, Cambridge, UK), and Santa Cruz (#sc-9021, Santa Cruz Biotechnology, Heidelberg, Germany) were tested as well as hybridoma cell supernatant (newly generated by immunizing mice against the peptide RRITRASALLDA, Abmart, Shanghai) and a newly generated antibody against recombinant SOCS1 (generated against recombinant SOCS1 by immunizing mice at Charles River, Chatillon-sur-Chalaronne, Ecully, France). On the next day, incubation with serum corresponding to the species of the secondary antibody followed. Incubation with the secondary antibody (goat anti-rabbit antibody or goat anti-mouse antibody) was performed for 30 min. After washing, sections were incubated with the chromogen (liquid diaminobenzidine and peroxide buffer) until a reaction was visible. Slides were counterstained with hematoxylin to provide nuclear and morphologic detail and mounted. Lung sections of *Socs1^−/−^* mice were used as a negative control.

### Flow Cytometry

The 2.5 × 10^5^ BMMs were harvested in PBS plus 2% (v/v) FCS and analyzed by FACSCanto flowcytometer gating on GFP positive cells (BD Bioscience, Heidelberg. Germany).

### Luciferase Activity Assay

The 2.5 × 10^5^ BMMs were lysed in Luciferase lysis buffer [1% (v/v) Triton X-100, 25 mM Glycyl-Glycine (pH 7.8), 15 mM MgSO4, 4 mM EGTA, and 1 mM DTT]. After injection of Luciferase assay buffer [25 mM K_3_PO_4_ (pH 7.8), 0.225 mM MgSO_4_, 0.08 mM EGTA, 2 mM ATP, 1 mM DTT, and 0.125 mM glycylglycine] to each well, activities in the lysates were measured using a luminometer (LUMIstar OPTIMA system, BMG LABTECH). Luminescent units are presented per microgram as determined by calorimetric Bradford assay using the Rotiquant reagent (Roth GmbH, Karlsruhe, Germany).

### Whole-Genome Expression Analysis

Total RNA from 2.5 × 10^5^ BMMs was isolated as described above. The quality of total RNA was checked by gel analysis using the total RNA Nano chip assay on an Agilent 2100 Bioanalyzer (Agilent Technologies GmbH, Berlin, Germany). Only samples with RNA index values greater than 8.5 were selected for expression profiling. RNA concentrations were determined using the NanoDrop spectrophotometer (NanoDrop Technologies, Wilmington, DE, USA). The laboratory work was done in the Genomics and Proteomics Core Facility at the German Cancer Research Center, Heidelberg, Germany (DKFZ). Biotin-labeled cRNA samples for hybridization on Illumina Mouse Sentrix-8 BeadChip arrays (Illumina, Inc.) were prepared according to Illumina’s recommended sample labeling procedure based on the modified Eberwine protocol (40). In brief, 300 ng total RNA was used for complementary DNA (cDNA) synthesis, followed by an amplification/labeling step (*in vitro* transcription) to synthesize biotin-labeled cRNA according to the Illumina^®^ Total Prep™ RNA Amplification Kit (Life Technologies). Biotin-16-UTP was purchased from Roche Applied Science, Penzberg, Germany. The cRNA was column purified according to TotalPrep RNA Amplification Kit, and eluted in 60 μl of water. Quality of cRNA was controlled using the RNA Nano Chip Assay on an Agilent 2100 Bioanalyzer and spectrophotometrically quantified (NanoDrop). Hybridization was performed at 58°C, in GEX-HCB buffer (Illumina, Inc.) at a concentration of 100 ng cRNA/μl, unsealed in a wet chamber for 20 h. Spike-in controls for low, medium, and highly abundant RNAs were added, as well as mismatch control and biotinylation control oligonucleotides. Microarrays were washed once in high temp wash buffer (Illumina, Inc.) at 55°C and then twice in E1BC buffer (Illumina, Inc.) at room temperature for 5 min (in between washed with ethanol at room temperature). After blocking for 5 min in 4 ml of 1% (wt/vol) Blocker Casein in phosphate buffered saline Hammarsten grade (Pierce Biotechnology, Inc., Rockford, IL, USA), array signals were developed by a 10 min incubation in 2 ml of 1 μg/ml Cy3-streptavidin (Amersham Biosciences, Buckinghamshire, UK) solution and 1% blocking solution. After a final wash in E1BC, the arrays were dried and scanned. Microarray scanning was done using an iScan array scanner. Data extraction was done for all beads individually, and outliers were removed when >2.5 median absolute deviation (MAD). All remaining data points were used for the calculation of the mean average signal for a given probe, and SD for each probe was calculated (ArrayExpress accession E-MTAB-4938). Data was processed using R, including log2 transformation of the data, significance (*p* ≤ 0.05), and fold change (log2 ≤ −1 or ≥1) filtered. Data were normalized to remove systematic variation and background subtraction. Pathway annotation was performed using the Protein Analysis through Evolutionary Relationships (PANTHER) classification system and transcription factor binding sites (TFBS) among the differentially regulated genes were analyzed using the overrepresentation analysis tool oPOSSUM.

### Enzyme-Linked Immunosorbent Assay

The 2.5 × 10^5^ CD11c^+^ cells were stimulated as indicated in 96-well plates in 200 μl RPMI supplemented with 10% (v/v) FCS and P/S. Supernatants were harvested and analyzed for cytokines by commercially available enzyme-linked immunosorbent assay (ELISA) kits for TNFα and IL-12p40 (BD Biosciences, Heidelberg, Germany). Cytokines were detected by measuring the absorbance at 490 nm with a 650 nm reference in a photometer (Sunrise reader, Tecan, Salzburg, Austria). Cytokine concentrations were calculated according to a standard dilution of the respective recombinant cytokines using Magellan V 5.0 software (Tecan, Salzburg, Austria).

### OVA Sensitization and Challenge

Mice were sensitized to ovalbumin (OVA) by three i.p. injections of 10 μg OVA (OVA grade VI; Sigma-Aldrich, Deisenhofen, Germany) adsorbed to 150 μg aluminum hydroxide (Imject Alum; Thermo, Rockford, IL, USA) on days 1, 14, and 21. Mice were exposed three times to an OVA (OVA grade V; Sigma-Aldrich) aerosol [1% (w/v) in PBS] on days 26, 27, and 28 to induce acute allergic airway inflammation (41). Sham sensitization and challenges were carried out using sterile PBS. Mice were sacrificed on day 29 by cervical dislocation under deep anesthesia. Eight animals per group were used, if not stated otherwise. Experiments were done at the Research Center Borstel under approval of the animal ethics committee from the Department of State, Kiel, Germany [permit no. V244-7224.121.3 (108-9/14)].

### Intratracheal IL-13 Instillation

Mice were anesthetized with isofluorane for 30 s and allowed to hang vertically with their mouths open, supported by a taut string placed under their canine teeth. Their tongues were gently withdrawn with a blunt forceps to keep them from swallowing. Twenty microliters of PBS with or without 5 μg IL-13 (#210-13, Peprotech, Hamburg, Germany) was applied onto the base of their tongues. When the mice had aspirated the applied solution, they were put on their site until they woke up. This intratracheal instillation was performed on days 1, 2, and 3. Mice were analyzed 24 h after the last treatment (permit no. 35-9185.81/G-35/16).

### Bronchoalveolar Lavage

Lungs were rinsed with 1 ml fresh, ice-cold PBS containing protease inhibitor (Roche, Basel, Switzerland) *via* a tracheal canula, and obtained cells were counted using a Neubauer chamber. Cytospins were prepared for each sample by centrifugation of 50 μl BAL fluid plus 150 μl of sterile PBS and subsequently stained with Diff-Quik (Medion Diagnostics, Duedingen, Switzerland). Cells were microscopically differentiated and classified as macrophages, neutrophils, eosinophils, or lymphocytes, using standard morphologic criteria (42).

### Cytokine and IgE Measurement

Levels of IL 4, IL-5, and IL-13 in serum were measured using an enhanced sensitivity cytometric bead array (CBA, Flex Set Kits; BD Biosciences, Franklin Lakes, NJ, USA), according to the manufacturer’s guidelines. IgE in serum was measured by ELISA. Briefly, 96-well high-binding ELISA plates (Greiner, Sigma-Aldrich, Deisenhofen, Germany) were coated with monoclonal anti-IgE antibodies (clone R35-72, BD Biosciences, Heidelberg, Germany) overnight. Serum samples were diluted in 1% (w/v) BSA in PBS/0.05% (v/v) Tween 20 and incubated overnight at 4°C. Afterward, plates were incubated with anti-IgE conjugated with HRP (clone 23G3, Southern Biotech, Birmingham, AL, USA) for 3 h at room temperature. For the colorimetric detection, TMB (Sigma) was used as a substrate. Absorbance was measured at 450 nm in ELISA reader (Infinite M200, Tecan) and IgE concentrations calculated according to standard curve.

### Primary Murine Tracheal Epithelial Cell Culture

The procedure used for isolation of murine tracheal epithelial cells was adapted from Davidson et al. (43). In brief, mice were killed by CO_2_ inhalation. Tracheas were removed, cut lengthways, washed in PBS, and transferred to collection media [1:1 mixture of DMEM and Ham’s F12 with 1% (v/v) penicillin–streptomycin]. Tracheas were then incubated at 4°C overnight in dissociation media [44 mM NaHCO_3_, 54 mM KCL, 110 mM NaCl, 0.9 mM NaH_2_PO_4_, 0.25 μM FeN_3_O_9_, 1 μM sodium pyruvate, pH 7.5, and supplemented with 1% (v/v) penicillin–streptomycin, 0.1 mg/ml DNaseI (#11284932001, Roche, Basel, Switzerland), and 1.4 mg/ml PronaseE (#P5147, Sigma, MO, USA)]. Enzymatic digestion was stopped by adding 20% FCS to the dissociation media. Epithelial cells were dissociated by gentle agitation followed by physical removal of the tracheas. Cells were then centrifuged at 1000 × *g* for 10 min at RT. Cell pellets were resuspended in culture medium [1:1 mixture of DMEM and Ham’s F12 with 1% penicillin-streptomycin, 5% FCS, and 120 U/l insulin (#12585014, ThermoFisher Scientific, Waltham, MA, USA)], and seeded in a 10 cm cell-culture dish for 2 h at 37°C. The supernatant was carefully taken off and centrifuged at 1000 × *g* for 10 min at RT. Cell pellets from two tracheas were resuspended in 200 μl culture medium and seeded in the inner well of a transwell (#CLS3470-48EA, Corning^®^ Costar^®^, Sigma, MO, USA) coated with human placenta collagen-IV (#C7521, Sigma, MO, USA). The outer well contained 600 μl of culture medium. After 7 days, medium from the inner well was removed and medium from the outer well was replaced with Ultroser G medium [1:1 mixture of DMEM and Ham’s F12 with 1% penicillin-streptomycin, 2% Ultroser G serum (#15950–017, Pall Corporation, Dreieich, Germany)]. After 30 days of culture, murine trachea epithelial cells were used for experiments. TER was measured using the Millicell^®^ electrical resistance system (ERS) (Millipore, Darmstadt, Germany).

### Preparation of Single-Cell Suspensions

For analysis of lung homogenate by qPCR, lungs were perfused through the right ventricle with PBS. Once lungs appeared white, they were removed and sectioned. Dissected lung tissue was then incubated with Liberase™ (100 μg/ml, #5401119001, Roche, Basel, Switzerland) and DNaseI (200 μg/ml, #11284932001, Sigma, MO, USA) at 37°C for 1 h. Digested lung tissue was gently disrupted by passage through a 19-G needle and afterward through a 70-μm pore size nylon cell strainer. Red blood cells were lysed using Red blood cell lysis buffer (eBioscience, CA, USA). CD11c^+^ or CD4^+^ T cells were isolated using the positive selection CD11c^+^ beads or the negative selection CD4^+^ T Cell Isolation Kit (Miltenyi Biotec, Bergisch Gladbach, Germany), respectively. Magnetically labeled cells were isolated *via* the autoMACS Separator (Miltenyi Biotec). For single cell suspension of splenocytes, spleens were dissected and treated as described above without enzymatic digestion.

### T Cell Differentiation

CD4^+^ T cells were isolated from a single cell suspension as described above. 1 × 10^5^ cells per well were plated in 96-well round bottom plate in 100 μl RPMI plus β-mercaptoethanol (50 μM) and stimulated with 4 μl anti-CD3 and anti-CD28-coated beads (#11456D, ThermoFisher Scientific, Waltham, MA, USA) + 20 ng/ml IL-2 (#402-ML-020, R&D Systems, Minneapolis, MN, USA), and either RPMI only (T0) or a Th2 differentiation solution consisting of 100 ng/ml IL-4 (#214-14, Peprotech, Hamburg, Germany), 10 μg/ml anti-IFNγ (#517903, BioLegend, San Diego, CA, USA), and 10 μg/ml anti-IL-12 (#505203, BioLegend, San Diego, CA, USA). Afterward, cells were incubated for 3 days at 37°C. On day 4, T cells were restimulated with a cell stimulation cocktail including PMA and ionomycin (#00-4970, eBioscience, CA, USA) for 3 h and RNA was extracted.

### NFκB p65 Activity Assay

NFκB activity was measured in 1 × 10^6^ cells of a lung homogenate by the TransAM™ NFκB p65 protein assay (Active Motif, Carlsbad, CA, USA), an ELISA-based method designed to specifically detect and quantify NFκB p65 subunit activation. As a positive control, Raji nuclear extract was used that was provided with the kit. Wild-type oligonucleotides were used as an internal specificity control. The assay was performed according to the manufacturer’s protocol and analyzed using a microplate absorbance reader (Sunrise reader, Tecan, Salzburg, Austria).

### Histopathological Analysis

Organs were fixed *via* a tracheal canula under constant pressure of 20 cm H_2_O using 4% (w/v) phosphate buffered paraformaldehyde overnight. Tissues were embedded in paraffin. For analysis of lung inflammation, 2 μm sections were stained with periodic acid–Schiff (PAS) or with hematoxylin and eosin (H&E), respectively.

### Statistics

All experiments were repeated three times unless stated otherwise. Data are shown as mean + SD. Statistical significance of comparison between two groups was determined by two-tailed unpaired Student’s *t*-test (for data sets following Gaussian distribution), Wilcoxon matched pairs test (for data sets not following Gaussian distribution), or two-way ANOVA including Bonferroni post-test (for multiple comparisons). All statistical analyses were done using GraphPad Prism (GraphPad 6.05, San Diego, CA, USA) software. Differences were considered significant at *p < 0.05, **p < 0.01, and ***p < 0.001.

## Results

### SOCS1ΔNLS Is Localized in the Cytoplasm

**Figure 1:**
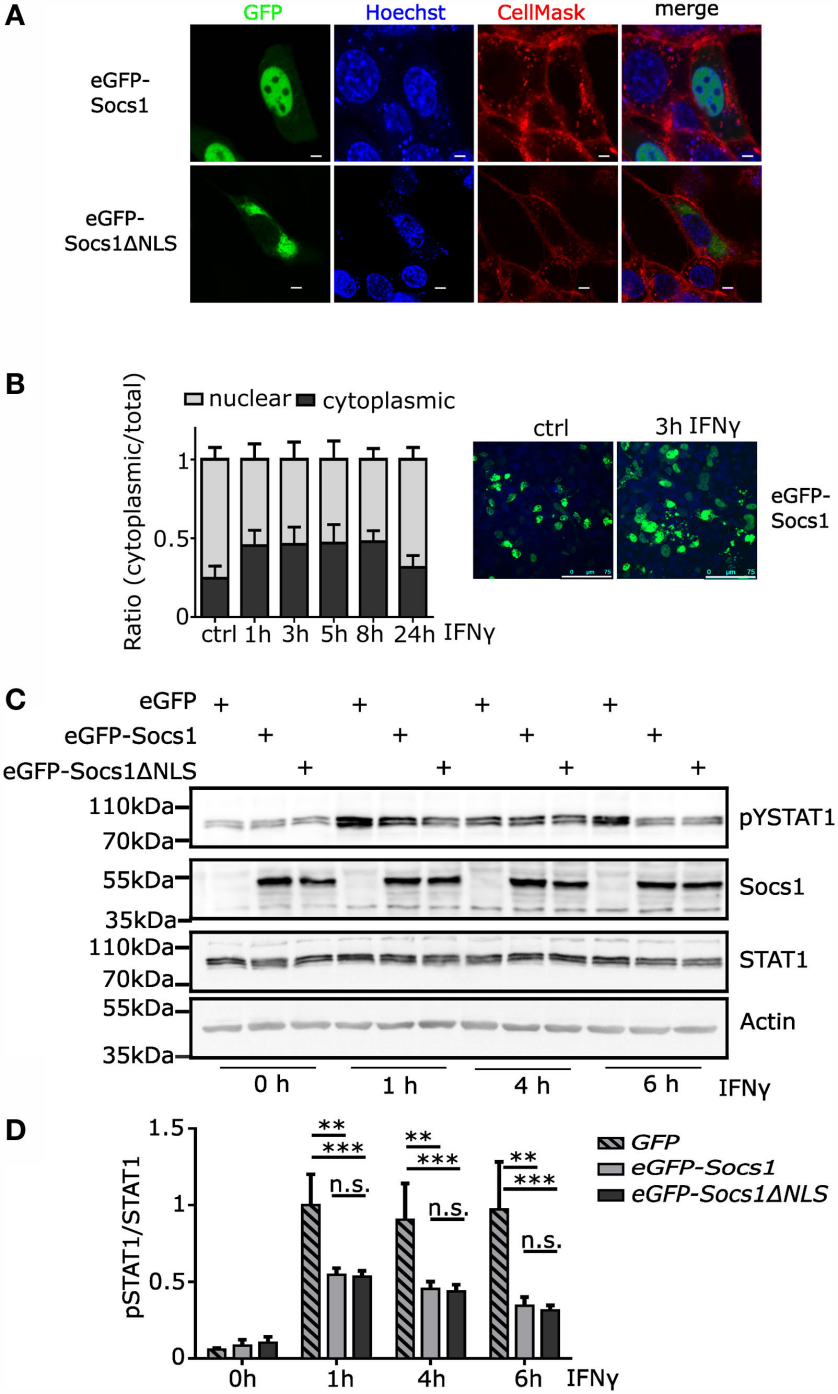
**Socs1ΔNLS is localized in the cytoplasm**. **(A)** NIH3T3 cells were transfected with the indicated eGFP-tagged plasmids and visualized by confocal microscopy. Nuclei were counterstained with Hoechst, and membranes were stained with CellMask™ dye. Scale bar, 5 μm. **(B)** NIH3T3 cells were transfected with *eGFP-Socs1* and stimulated with IFNγ (50 ng/ml) as indicated. A *z*-stack was recorded and the ratio of the fluorescence in the cytoplasm versus the fluorescence in the nucleus was measured using ImageJ by setting an ROI around either the cytoplasm or the nucleus (*n* = 3 with 20–50 cells each). Scale bar, 75 μm. **(C)** Western blot analysis of tyrosine phosphorylated STAT1. Raw264.7 cells were transfected with *eGFP, eGFP-Socs1*, or *eGFP-Socs1ΔNLS* and stimulated with IFNγ (50 ng/ml) as indicated. Protein extracts were stained for pY-STAT1 (Tyr701), SOCS1, STAT1, and β-actin. Quantification using ImageJ is shown in **(D)** (*n* = 3, mean + SD, two-way ANOVA including Bonferroni post-test).

It has been shown that SOCS1 is able to translocate into the cell nucleus due to a functional NLS localized between the SH2 domain and the SOCS-box (amino acid 159–173) (33, 34). Confirming these results with murine SOCS1 constructs, NIH3T3 cells transiently transfected with murine *eGFP-Socs1* showed nuclear localization of the GFP-tagged protein. In contrast, eGFP-SOCS1ΔNLS, in which the NLS has been replaced by the murine SOCS3 sequence (33), was localized more in the cytoplasm (Figure 1A). Cells transfected with *Socs1wt* were also stimulated with IFNγ. Upon stimulation enhanced fluorescence in the cytoplasm could be observed, suggesting that SOCS1 is partly translocating out of the nucleus to inhibit signaling in the cytoplasm (Figure 1B). To verify that SOCS1ΔNLS is still functional in the cytoplasm, inhibition of IFNγ signaling was analyzed by Western Blotting. Therefore, the murine macrophage cell line Raw264.7 was transiently transfected with *eGFP, eGFP-Socs1*, or *eGFP-Socs1ΔNLS*. Tyrosine phosphorylation of STAT1 was examined upon treatment with IFNγ 1–6 h post-transfection (Figures 1C,D). Already 1 h after IFNγ treatment, *eGFP-Socs1* or *eGFP-Socs1ΔNLS* transfected cells showed lower levels of phosphorylated STAT1 (59 or 57% as compared to *eGFP* transfected cells 1 h after IFNγ stimulation). Importantly, we could not observe any differences in phosphorylated STAT1 levels between *eGFP-Socs1* and *eGFP-Socs1*Δ*NLS-*transfected cells. Data suggest that both SOCS1 and SOCS1ΔNLS were effectively inhibiting IFNγ-induced STAT1 tyrosine phosphorylation, which occurs at the level of receptor activation.

### Generation and Characterization of Mice Expressing Non-Nuclear SOCS1ΔNLS

**Figure 2:**
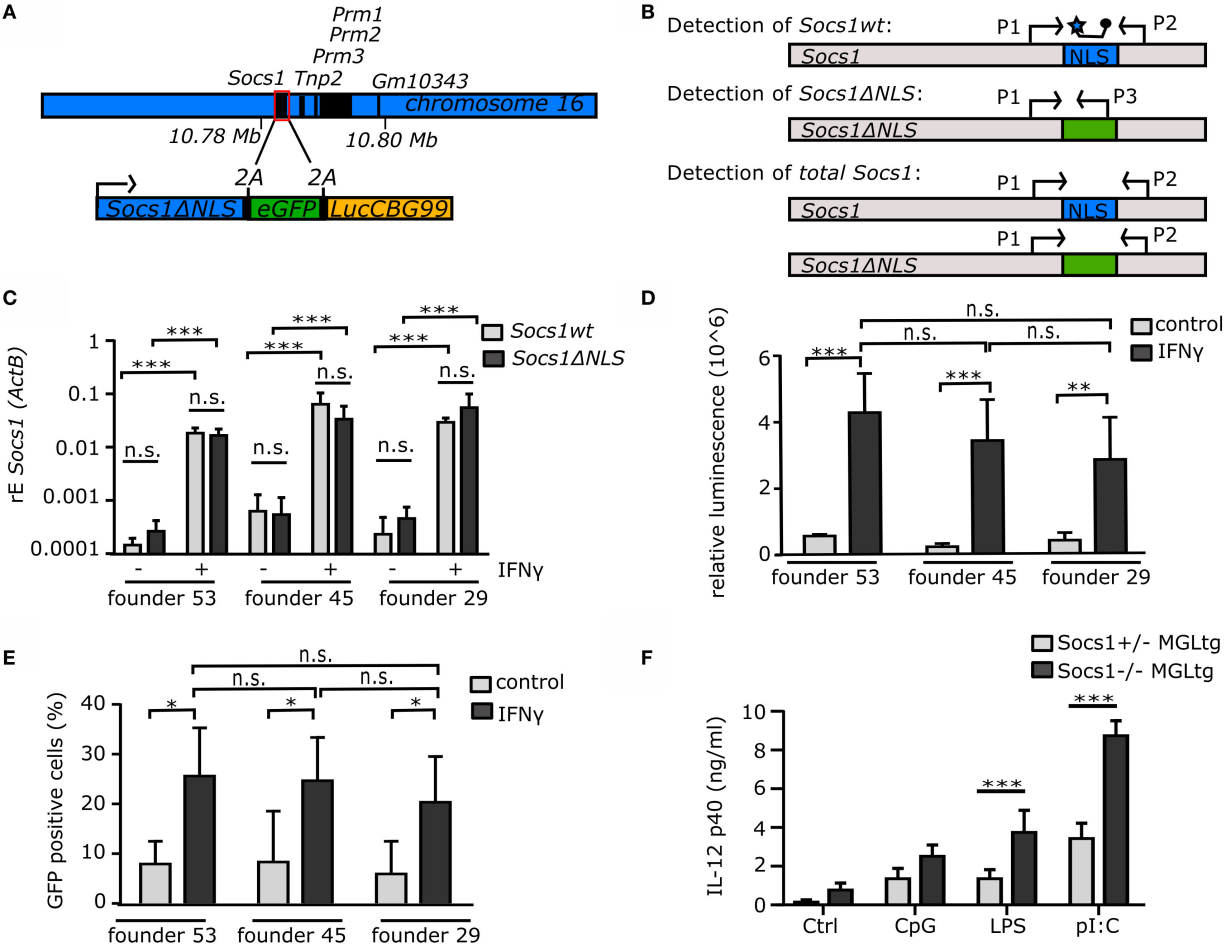
**Generation of Socs1ΔNLS transgenic mice**. **(A)** Schematic drawing of the bacterial artificial chromosome (BAC) consisting of a mutated *Socs1* locus containing *Socs1ΔNLS, eGFP*, and *LuciferaseCBG99*. 2A sequences result in cleavage of the polyprotein. **(B)** qPCR strategy to specifically detect *Socs1 wildtype* (wt) using the primer P1 and P2 in combination with a labeled probe within the NLS, *Socs1ΔNLS* using the reverse primer P3 in the NLS or total *Socs1* using the primer P1 and P2. BMMs of *Socs1^+/+^MGL^tg^* mice of three different founders were stimulated with IFNγ (50 ng/ml) for 24 h and analyzed for **(C)** mRNA expression of *Socs1 wt* and *Socs1ΔNLS*, **(D)** luciferase activity, and **(E)** the percentage of GFP positive cells (*n* = 4–5, mean + SD, two-way ANOVA including Bonferroni post-test). **(F)** CD11c^+^ cells were isolated from lung homogenate and stimulated with CpG (1 μM), LPS (100 ng/ml), and pI:C (10 μg/ml) for 24 h. IL-12p40 protein levels were measured by ELISA (*n* = 3–5, mean + SD, two-way ANOVA including Bonferroni post-test).

To analyze the function of SOCS1 in the cell nucleus *in vivo*, transgenic mice were established using a BAC containing a mutated *Socs1* locus with non-nuclear *Socs1ΔNLS*, e*GFP*, and *LuciferaseCBG99*, termed MGL (Figure 2A). 2A peptide sequences between the protein coding regions result in three separate proteins. Thereby, 21 amino acids remain at the C-terminus of SOCS1ΔNLS. The combined expression of GFP and luciferase together with SOCS1 allows using these mice as reporter mice as well. Quantitative real-time PCR was established to confirm mRNA expression of *Socs1ΔNLS* in bone marrow-derived macrophages (BMMs) from BAC transgenic mice (Figure 2B). To exclude founder-specific effects due to different integration sites of the BAC, stable expression and regulation of the mutated *Socs1* locus was examined in different founders. Therefore, offsprings of the founders #53, #45, and #29 were analyzed with respect to the expression of *Socs1* and *Socs1ΔNLS* in BMMs upon stimulation with IFNγ for 24 h using the qPCR strategy as described in Figure 2B. After stimulation with IFNγ, mRNA expression of both *Socs1* and *Socs1ΔNLS* were induced to a similar amount in all three founders (Figure 2C). We observed no differences in expression levels of *Socs1wt* and *Socs1ΔNLS*. Due to reporter functions of the mutated *Socs1* locus, luciferase assay was performed in BMMs after stimulation with IFNγ, showing similar luciferase activity in BMMs for the three founders (Figure 2D). In addition, GFP positive BMMs were analyzed by flow cytometry (Figure 2E). There was an increase in the percentage of GFP-positive cells after stimulation with IFNγ, and this was similar for founders #53 (26% ± 9.6), #45 (25% ± 8.7), and #29 (21% ± 9.2). Taken together, all three founders showed similar expression levels of the mutated *Socs1* locus. Mutant *Socs1* expression was also in the same range as *Socs1 wild-type (wt)* mRNA, suggesting that different integration sites did not influence expression of the BAC. Thus, we had no indication for a founder specific effect and, therefore, further experiments were performed using founder #53 if not stated otherwise.

### Functional Impairment of NFκB Inhibition in *Socs1^−/−^MGL^tg^* Mice

To analyze the function of SOCS1 in the cell nucleus, *Socs1MGL^tg^* mice were mated with *Socs1^+/−^* mice to generate *Socs1^−/−^MGL^tg^* mice, expressing *Socs1ΔNLS* in an otherwise *Socs1*-deficient background. In order to show that *Socs1^−/−^MGL^tg^* mice indeed lack SOCS1 in the cell nucleus, we tried staining of endogenous SOCS1 by immunohistochemistry. However, we could not find a sufficiently specific antibody that was not staining sections from *Socs1^−/−^* mice (including newly generated antibodies). Therefore, we decided to do a functional approach to verify non-nuclear expression of *Socs1MGL*. It has been reported that nuclear SOCS1 limits NFκB signaling by degradation of the NFκB subunit p65 (38). To examine whether NFκB signaling is altered in *Socs1^−/−^MGL^tg^* mice, CD11c^+^ cells were isolated from lungs and stimulated *ex vivo* with TLR agonists. Stimulation of CD11c^+^ cells from *Socs1^−/−^MGL^tg^* mice with CpG-DNA, LPS, and pI:C for 24 h led to an increased protein expression of IL-12p40 as compared to CD11c^+^ cells from *Socs1^+/−^MGL^tg^* mice (Figure 2F). The same could be shown in CD11c*^+^* cells isolated from spleens (Figure S1 in Supplementary Material). Data suggest sustained NFκB activation in *Socs1^−/−^MGL^tg^* mice that was confirmed by transcription factor binding assay for p65 (Figure S1 in Supplementary Material). We did not detect differences regarding TNFα protein levels (Figure S1 in Supplementary Material), which is in full accordance with previous findings (38) showing that only a subset of NFκB dependent genes is altered in *Socs1^−/−^MGL^tg^* mice. In contrast, IL-12p40 induction that needs prolonged binding of NFκB to its promoter (44) was sensitive to SOCS1-induced NFκB inhibition. The results entirely phenocopy *in vitro* data using non-nuclear SOCS1ΔNLS, suggesting that *Socs1^−/−^MGL^tg^* mice functionally lack SOCS1 in the cell nucleus.

### *Socs1^−/−^MGL^tg^* Mice Survive the Early Lethal Phenotype as Compared to *Socs1^−/−^* Mice

**Figure 3:**
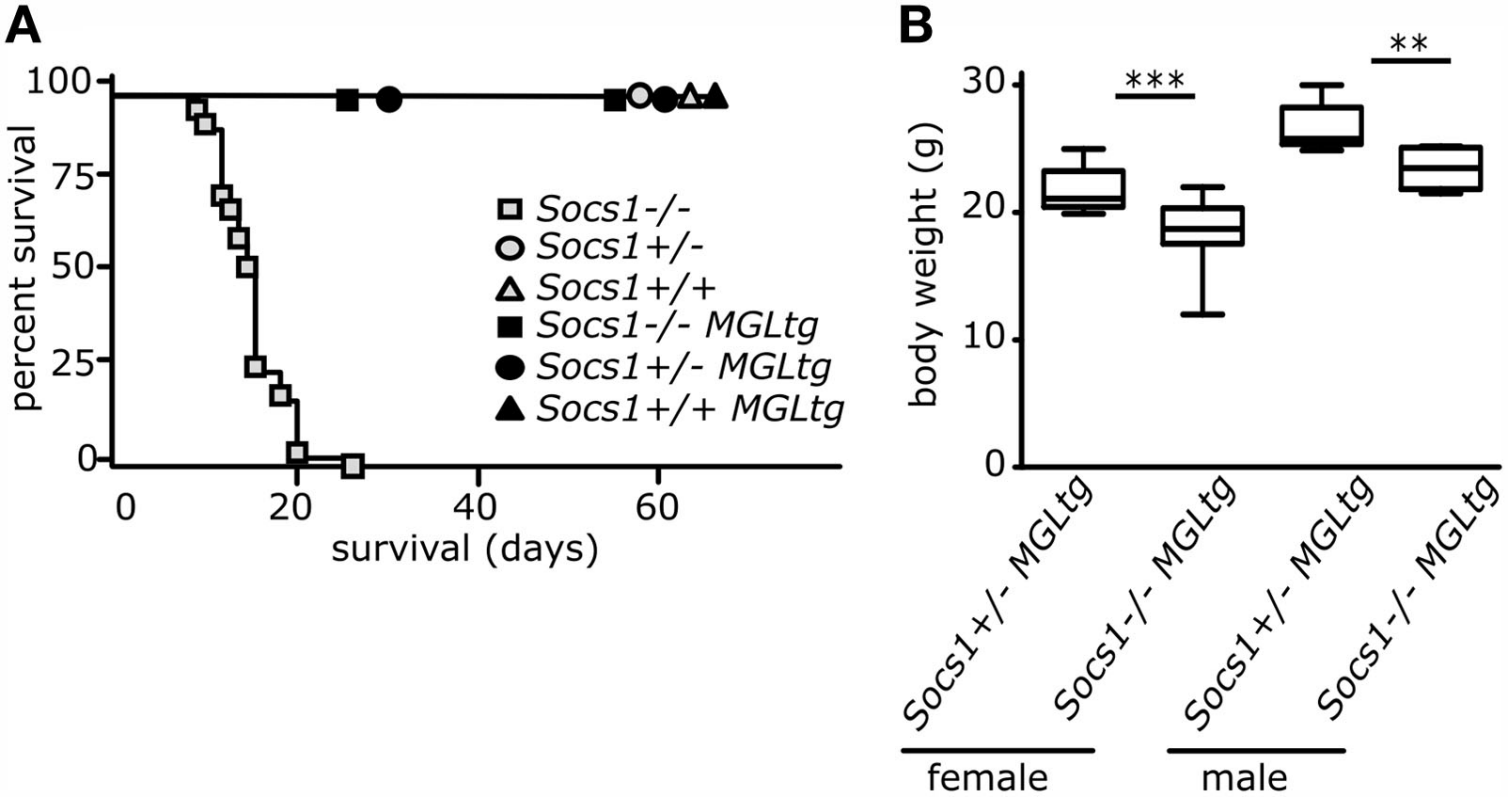
***Socs1^−/−^MGL^tg^* mice survive the early lethal phenotype of *Socs1^−/−^* mice, but show a reduced body weight**. **(A)** After mating *Socs1*^+/−^ to *Socs1*^+/−^*MGL^tg^* mice, survival of offsprings was recorded to the age of 60 days (*n* = 28–92 mice per genotype). **(B)** Mouse body weight analysis (*n* = 10–15 8-week-old mice per gender and genotype, mean + SD, Wilcoxon matched pairs test).

*Socs1^−/−^* mice die within 2–3 weeks due to multiorgan inflammation (24–26). In contrast, *Socs1^−/−^MGL^tg^* mice survived and showed no early lethality up to 60 days (Figure 3A), suggesting that lack of *Socs1wt* is rescued by *Socs1ΔNLS*. *Socs1*^+/−^ mice also showed normal survival indicating that one allele of *Socs1* is sufficient for rescue of the severe knockout phenotype. In a small cohort (*n* = 4), survival of *Socs1^−/−^MGL^tg^* mice was recorded for an extended period (Figure S2A in Supplementary Material). Up to 38 weeks, *Socs1^−/−^MGL^tg^* mice appeared healthy without overt abnormalities. Although lethality was rescued in *Socs1^−/−^MGL^tg^* mice, the mice showed reduced body weight both for female and male 8-week-old mice (Figure 3B) suggesting that lack of nuclear SOCS1 results in partial functional impairment. In the long-term survival cohort, *Socs1^−/−^MGL^tg^* mice showed slightly reduced body weight as well (Figure S2 in Supplementary Material).

### Similar mRNA Expression Levels, Protein Levels, and Protein Half-Life of SOCS1 and SOCS1ΔNLS

**Figure 4:**
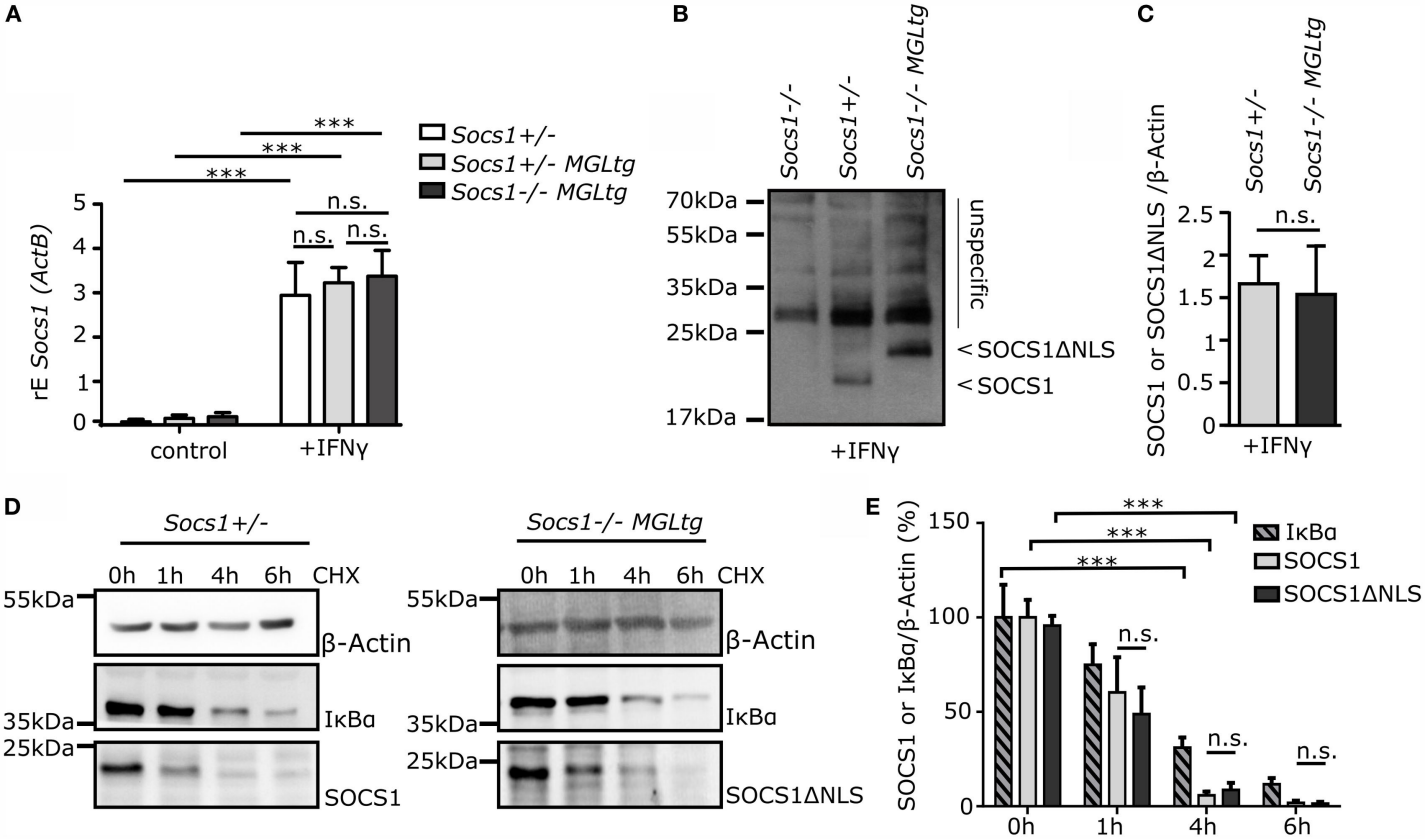
**Similar mRNA expression levels, protein levels, and protein half-life of SOCS1 and SOCS1ΔNLS. (A)** Total *Socs1* mRNA expression levels in BMMs of *Socs1*^+/−^, *Socs1^+/−^MGL^tg^*, and *Socs1^−/−^MGL^tg^* mice upon stimulation with IFNγ (50 ng/ml) for 24 h normalized to Actin (ActB) expression (*n* = 4, mean + SD, two-way ANOVA including Bonferroni post-test). **(B)** Detection of SOCS1 protein in *Socs1^−/−^, Socs1*^+/−^, and *Socs1^−/−^MGL^tg^* mice upon stimulation with IFNγ (50 ng/ml) for 6 h using anti-SOCS1 hybridoma cell supernatant. Arrows indicate band for SOCS1 and SOCS1ΔNLS. **(C)** Quantification of SOCS1 protein for *Socs1*^+/−^ mice and of SOCS1ΔNLS protein for *Socs1^−/−^MGL^tg^* mice compared to β-actin was done using ImageJ (*n* = 10–13, mean + SD, Student’s *t*-test). **(D)** Protein stability was assessed performing a cycloheximide (CHX) chase. CHX (100 μg/ml) was added at 6 h poststimulation with IFNγ and BMMs were lysed at the indicated timepoints and analyzed for SOCS1 expression by Western Blotting. **(E)** SOCS1 and SOCS1ΔNLS protein expression was normalized to β-actin in order to calculate the percentage of remaining protein relative to its expression prior to the addition of CHX. IkBα was used as a control for effective CHX treatment (*n* = 3–5, mean + SD, two-way ANOVA including Bonferroni post-test).

To verify that BMMs of *Socs1^−/−^MGL^tg^* mice have similar expression levels of total *Socs1* (both *Socs1* and *Socs1ΔNLS*) as compared to BMMs of *Socs1*^+/−^ and *Socs1*^+/−^*MGL^tg^* mice, cells were stimulated with IFNγ for 24 h and qPCR was performed to detect mRNA of total *Socs1* using the qPCR strategy as described in Figure 2B. Similar expression of total *Socs1* mRNA could be verified in BMMs of *Socs1^−/−^MGL^tg^, Socs1*^+/−^, and *Socs1*^+/−^*MGL^tg^* mice (Figure 4A). For further analysis, we generated a new SOCS1 antibody. This antibody did not detect a band with the expected molecular weight for SOCS1 in lysates of BMMs of *Socs1*^−/−^mice (Figure 4B), thus proving specificity in Western Blot analysis. We confirmed expression of SOCS1 and SOCS1ΔNLS protein in lysates of BMMs of *Socs1*^+/−^ and *Socs1^−/−^MGL^tg^* mice stimulated with IFNγ for 6 h. The higher molecular weight of SOCS1ΔNLS likely resulted from the additional 21 amino acids after cleavage of the 2A sequence. In Figure 4C, quantification was done normalized to β-actin expression. Comparable protein levels for SOCS1 and SOCS1ΔNLS were detected (Figure 4B). The NLS of SOCS1 (RRMLGAPLRQRRVR) resembles a bipartite NLS composed of two basic stretches. Since the basic amino acid lysine is important for marking proteins for the ubiquitin proteasome pathway (45, 46), we addressed the question whether exchanging the NLS with the SOCS3 counterpart might alter protein half-life. We, therefore, performed a cycloheximide (CHX) chase experiment (Figures 4D,E). Six hours post stimulation with IFNγ, CHX was added to block nascent protein synthesis. Already after 4 h of CHX treatment, there was only 4% of the SOCS1 protein and 12% of the SOCS1ΔNLS protein remaining. In summary, we did not observe alteration of mRNA expression levels, protein levels, or protein half-life upon mutating SOCS1 into SOCS1ΔNLS.

### Functional Regulation of Canonical IFNγ Signaling in *Socs1^−/−^MGL^tg^* Mice

**Figure 5:**
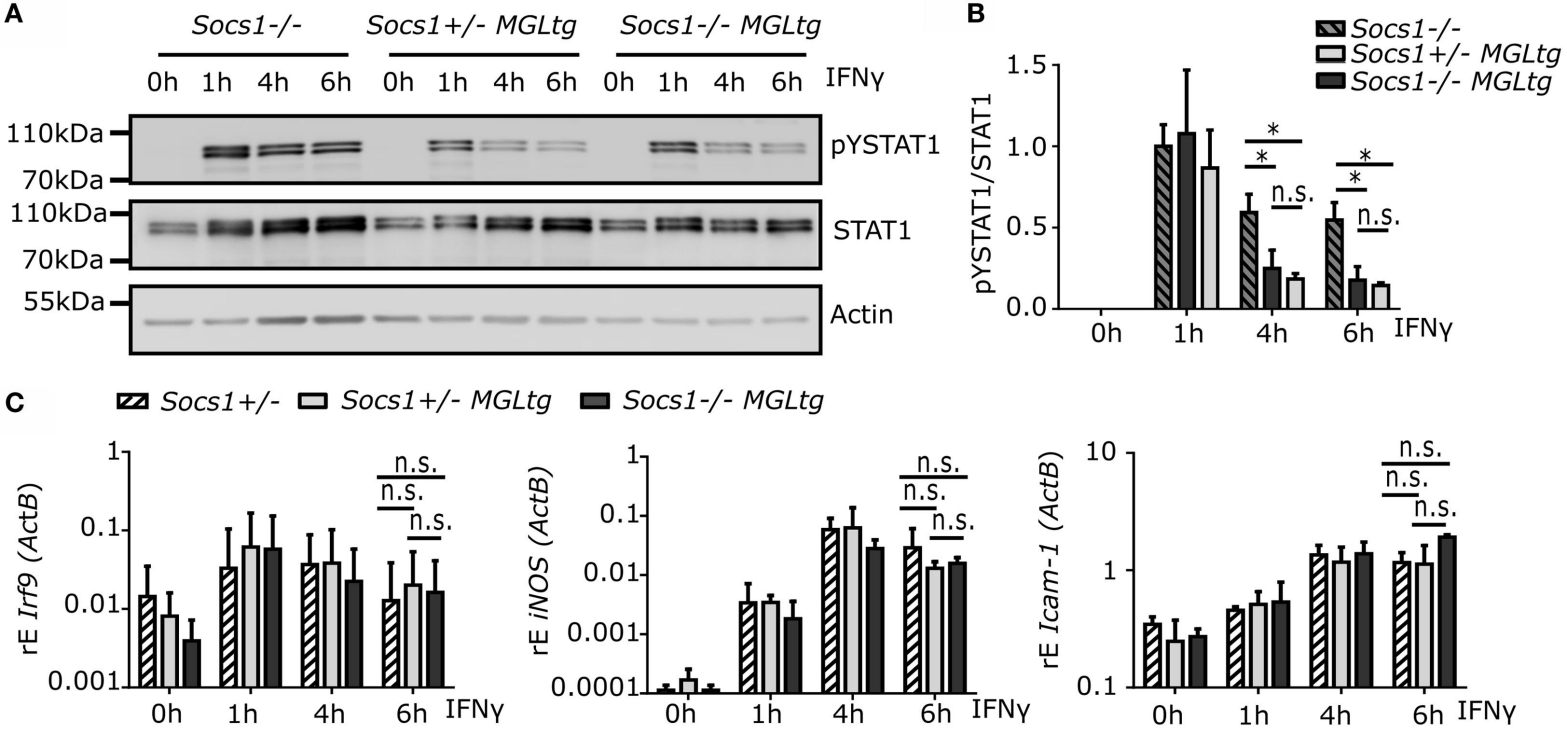
**Canonical IFNγ signaling is not altered in *Socs1^−/−^MGL^tg^* mice**. **(A)** Western blot analysis of tyrosine phosphorylated STAT1 (Tyr701). STAT1 and β-actin were used as loading controls. Protein extracts were prepared from BMMs of *Socs1^−/−^, Socs1*^+/−^*MGL^tg^*, and *Socs1^−/−^MGL^tg^* mice that were treated with IFNγ (50 ng/ml) as indicated. Quantification using ImageJ is shown in **(B)** (*n* = 4). **(C)** mRNA expression of interferon target genes *iNOS, Irf9*, and *Icam-1* are shown normalized to Actin (ActB) expression in BMMs of *Socs*^+/−^, *Socs1*^+/−^*MGL^tg^*, and *Socs1^−/−^MGL^tg^* mice treated with IFNγ (50 ng/ml) for 1–6 h (*n* = 4). Mean + SD is presented for each group and significance was assessed using two-way ANOVA including Bonferroni post-test.

*Socs1^−/−^MGL^tg^* mice survived the early lethal phenotype of *Socs1^−/−^* mice (Figure 3A). As it is known that *Socs1^−/−^* can be rescued by the administration of anti-IFNγ antibodies in the neonatal period or by using *Socs1^−/−^IFNγ^−/−^* mice (47, 48), we hypothesized that canonical IFNγ signaling is not altered in *Socs1^−/−^MGL^tg^* mice. To test this hypothesis, tyrosine phosphorylation of STAT1 was examined in BMMs upon treatment with IFNγ for 1–6 h (Figure 5A). IFNγ signaling was prolonged in *Socs1^−/−^* mice as shown by the sustained levels of phosphorylated STAT1. There was a decline in phosphorylated STAT1 levels for both *Socs1*^+/−^*MGL^tg^* mice (to 20%) and *Socs1^−/−^MGL^tg^* mice (to 22%) already 4 h after IFNγ stimulation as compared to 58% for *Socs1^−/−^* mice (Figures 5A,B). No significant differences were observed between *Socs1*^+/−^*MGL^tg^* and *Socs1^−/−^MGL^tg^* mice. Analyzing mRNA expression levels of classical IFNγ target genes, both *iNOS* and *Irf9* were induced upon stimulation with IFNγ to a similar extent in *Socs1*^+/−^*MGL^tg^* and *Socs1^−/−^MGL^tg^* mice (Figure 5C). Of note, the expression levels were also similar to *Socs1*^+/−^ mice, arguing against gene dosage effects. This allows interpretation of the data from *Socs1^−/−^MGL^tg^* mice with regards to lack of nuclear SOCS1 and not altered concentration of cytoplasmic SOCS1. There was a minor, but non-significant, increase in the expression level of *Icam-1* after 6 h of stimulation in BMMs of *Socs1^−/−^MGL^tg^* mice. These findings indicate that SOCS1ΔNLS was still able to regulate cytoplasmic signaling pathways and that canonical IFNγ signaling was not altered in *Socs1^−/−^MGL^tg^* mice.

### Differential Expression of a Subset of Non-Canonical IFNγ Target Genes by *Socs1^−/−^MGL^tg^* Mice

**Figure 6:**
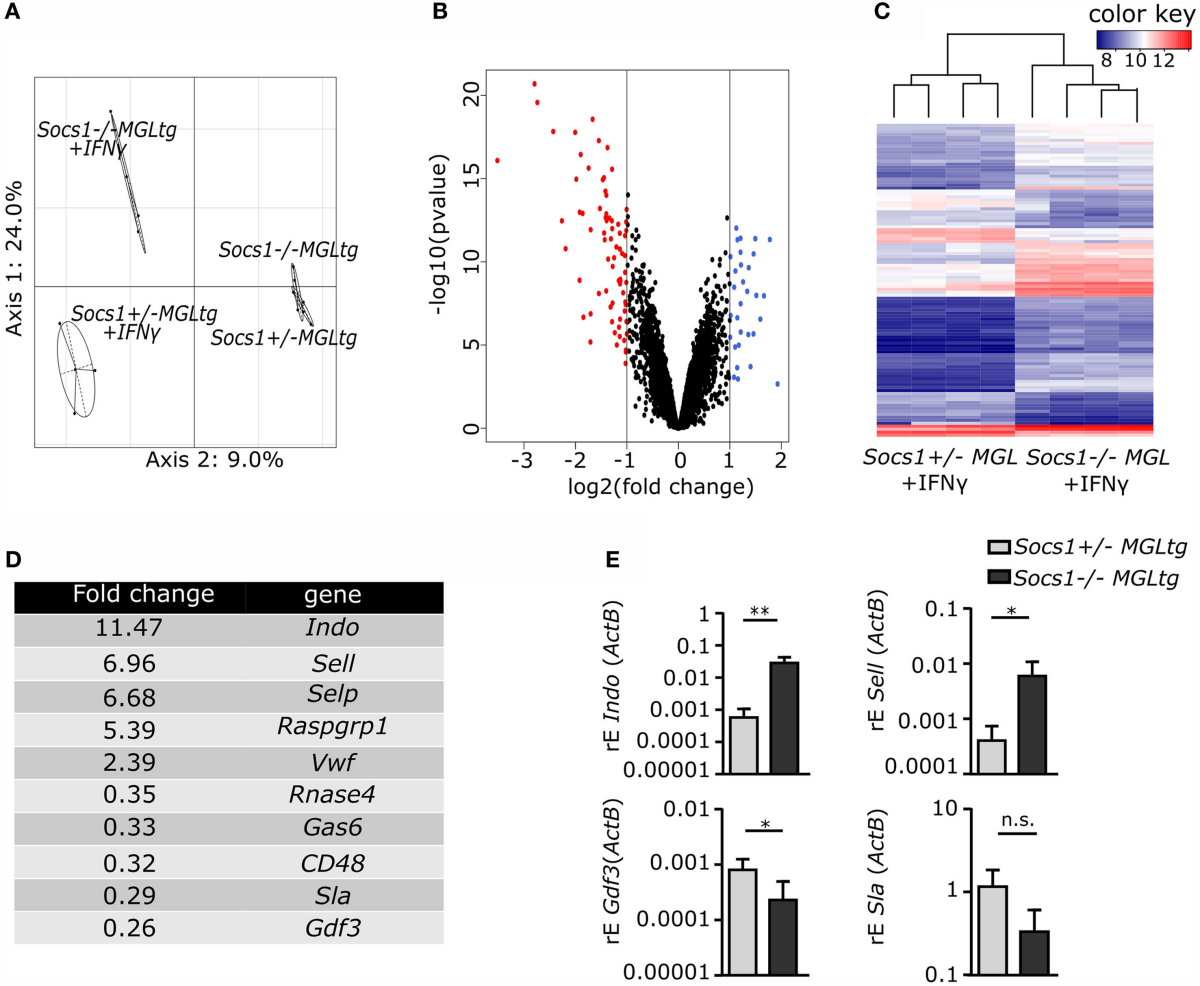
**Subset of non-classical IFNγ target genes is differentially regulated in *Socs1^−/−^MGL^tg^* mice**. BMMs were stimulated with IFNγ for 24 h, RNA was extracted and subjected to whole-genome expression analysis **(A)** principal component analysis (PCA). The two principal components and their fraction of the overall variability of the data (%) are shown on the *x*-axis and the *y*-axis. Clusters of experiments are circled (95% confidence interval ellipse) and annotated as *Socs1*^+/−^*MGL^tg^* ctrl and *Socs1^−/−^MGL^tg^* ctrl for untreated samples and *Socs1*^+/−^*MGL^tg^* + IFNγ and *Socs1^−/−^MGL^tg^* + IFNγ for IFNγ-stimulated samples. **(B)** Volcano plot showing the genes that were differentially expressed between BMMs of *Socs1*^+/−^*MGL^tg^* and *Socs1^−/−^MGL^tg^* mice. Only significant values (*p* ≤ 0.05) were considered showing a log2-fold change ≤−1 (red points) or ≥1 (blue points). **(C)** Heat map visualizing hierarchical clustering analysis based on the expression levels of genes that are differentially expressed between BMMs of *Socs1*^+/−^*MGL^tg^* and *Socs1^−/−^MGL^tg^* mice. Red indicates higher expression and blue indicates lower expression of the corresponding gene in *Socs1^−/−^MGL^tg^* mice, respectively (*n* = 4). **(D)** 10 most prominently up- and downregulated genes according to their fold change. **(E)** Quantitative RT-PCR was performed using RNA from BMMs stimulated with IFNγ for 24 h (*n* = 3, mean + SD, Student’s *t*-test).

To support the hypothesis that canonical IFNγ signaling is not altered in *Socs1^−/−^MGL^tg^* mice, whole-genome expression analysis was performed. Therefore, BMMs were stimulated with IFNγ for 24 h and RNA was extracted and subjected to whole-genome expression analysis. 1097 genes were differentially regulated between untreated and IFNγ stimulated cells, but only 86 genes were differentially regulated between BMMs of *Socs1^−/−^MGL^tg^* mice and *Socs1*^+/−^*MGL^tg^* mice. To analyze combinatorial patterns in an unbiased fashion, principal component analysis (PCA) was performed. Analysis of untreated BMMs of both genotypes revealed close correlation, whereas IFNγ treated BMMs of *Socs1^−/−^MGL^tg^* mice and *Socs1*^+/−^*MGL^tg^* mice were found to be more separated (Figure 6A). Most differentially regulated genes were induced rather than repressed in *Socs1^−/−^MGL^tg^* mice (Figures 6B,C). The top 10 differentially regulated genes (Figure 6D) included significantly higher expressed genes in BMMs of *Socs1^−/−^MGL^tg^* mice such as *Indoleamine 2,3-Dioxygenase 1* (*Indo*, 11.47-fold) and *SelectinL* (*Sell*, 6.69-fold) as well as significantly lower expressed genes in BMMs of *Socs1^−/−^MGL^tg^* mice such as *Src-Like-Adaptor* (*Sla*, 0.29-fold) and *Growth Differentiation Factor 3* (*Gdf3*, 0.26-fold). Those genes were confirmed by qPCR to be differentially regulated in *Socs1^−/−^MGL^tg^* mice (Figure 6E). Importantly, no canonical IFNγ target genes were differentially regulated in *Socs1^−/−^MGL^tg^* mice. Of note, we found 38 genes involved in NFκB signaling to be upregulated in BMMs of *Socs1^−/−^MGL^tg^* mice and 16 downregulated ones. Pathway annotation was performed using the PANTHER classification system among the 86 genes differentially regulated between IFNγ treated BMMs of *Socs1*^+/−^*MGL^tg^* and *Socs1^−/−^MGL^tg^* mice (Table 1). Instead of IFNγ signaling pathway, we found TLR and TNF signaling pathways to be dysregulated. For TLR signaling pathway, three genes assigned to the signaling pathway were significantly higher expressed and five were significantly lower expressed in BMMs of *Socs1^−/−^MGL^tg^* mice. For TNF signaling pathway, five genes assigned to the signaling pathway were significantly higher expressed, whereas only one was significantly lower expressed in BMMs of *Socs1^−/−^MGL^tg^* mice, arguing for a general induction of the pathway. Moreover, TFBS among the differentially regulated genes were analyzed using the overrepresentation analysis tool oPOSSUM (Table 2). TFBS for CTCF, IRF2, and NFκB were overrepresented among the differentially regulated genes in *Socs1^−/−^MGL^tg^* mice with 21, 8, and 56 genes, respectively. STAT1 as classical transcription factor for IFNγ signaling was not overrepresented among the differentially regulated genes, strengthening the hypothesis of functional regulation of canonical IFNγ signaling by Socs1ΔNLS in *Socs1^−/−^MGL^tg^* mice. In summary, whole genome expression analysis revealed a small subset of non-canonical IFNγ-regulated genes that were differentially regulated in *Socs1^−/−^MGL^tg^* mice with an overrepresentation of TFBS for NFκB.

**Table 1:** **Pathway annotation [protein analysis through evolutionary relationships (PANTHER) classification system]**.

Pathway ID	Input/background	Induced genes	Repressed genes	*p*-Value
TLR signaling pathway	8/101	*CD40, Cxcl10, Jun*	*Ccl3, Ccl4, Ccl5, Tlr7, Tlr8*	0.00129
TNF signaling pathway	6/109	*Socs3, Fas, Tnfaip3, Cxcl10, Jun*	*Ccl5*	0.056

*Differentially expressed genes in Socs1^−/−^MGL^tg^ mice upon IFNγ stimulation showing a log2-fold change (≤−1 or ≥1) and *p* ≤ 0.05 were analyzed to classify and identify gene functions*.

**Table 2:** **oPOSSUM analysis of overrepresented transcription factor binding sites**.

TFBS	Input/background	Class	Family	*z*-score	Fischer score
CTCF	21/86	Zinc coordinating	ββα zinc finger	14.88	5.01
IRF2	8/86	Winged helix-turn-helix	IRF	14.343	4.42
NFκB	56/86	Ig fold	Rel	12.24	2.28

*Differentially expressed genes in Socs1^−/−^MGL^tg^ mice upon IFNγ stimulation showing a log2-fold change (≤−1 or ≥1) and *p* ≤ 0.05 were uploaded to identify overrepresented transcription factor binding sites among those genes. Transcription factors enriched in the corresponding gene set were CTCF, IRF2, and NFkB*.

### *Socs1^−/−^MGL^tg^* Mice Spontaneously Develop Low-Grade Inflammation in the Lung

**Figure 7:**
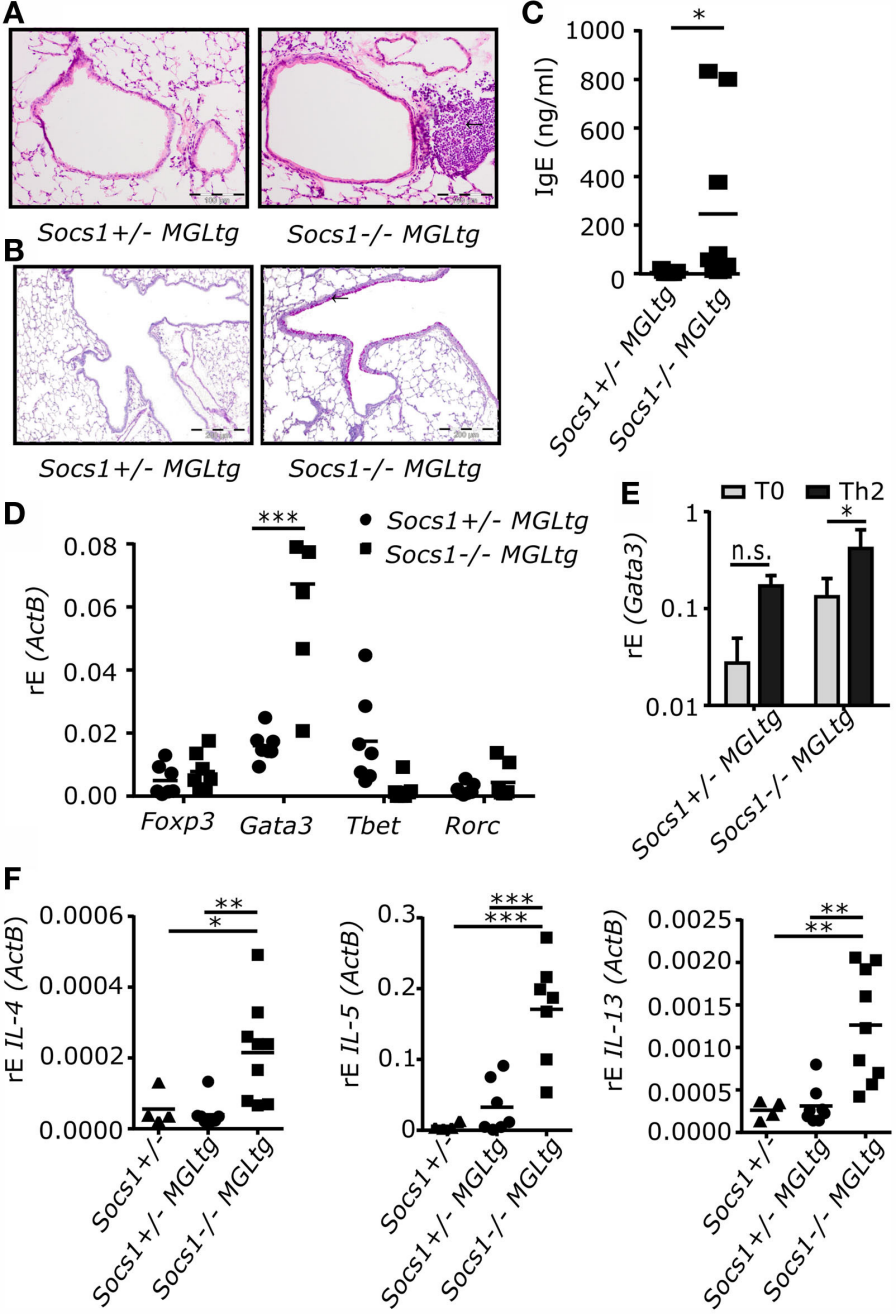
***Socs1^−/−^MGL^tg^* mice show low-grade inflammation in the lung**. **(A)** H&E-stained airway cross sections. Arrow highlighting infiltrating cells. Scale bar, 100 μm. One lung section per mouse was analyzed. **(B)** Periodic acid–Schiff (PAS) stained airway cross sections. Arrow highlighting PAS positive cells. Scale bar, 200 μm. **(C)** IgE concentration in serum (*n* = 9, mean + SD, Wilcoxon matched pairs test). **(D)** Expression levels of *Foxp3, Gata3, Tbet*, and *Rorc* in sorted *CD4^+^* T cells in the lung of both founder #53 and #29 (*n* = 7–11, mean + SD, two-way ANOVA including Bonferroni post-test). **(E)** Naive CD4^+^ T cells were differentiated under T_0_ conditions (RPMI only) or under Th2 conditions (100 ng/ml IL-4, 10 μg/ml anti-IFNγ, and 10 μg/ml anti-IL-12p40) for 3 days and restimulated using PMA and ionomycin. Expression of Gata3 was examined by qPCR (*n* = 3, mean + SD, two-way ANOVA including Bonferroni post-test). **(F)** Expression levels of *IL-4, IL-5*, and *IL-13* in total lung homogenates of both founder #53 and #29 (*n* = 7–11, mean + SD, one-way ANOVA including Bonferroni post-test).

Although inhibition of IFNγ signaling by SOCS1ΔNLS was still functional in *Socs1^−/−^MGL^tg^* mice, gene expression analysis indicated that differences due to lack of SOCS1 in the cell nucleus were present. We closely analyzed *Socs1^−/−^MGL^tg^* mice for disease symptoms. Histopathological analysis revealed low-grade inflammation in lung and liver in a significant number of *Socs1^−/−^MGL^tg^* mice (Table S2 in Supplementary Material). However, no differences were observed in serum AST and ALT levels (Figure S3 in Supplementary Material). We, therefore, focused on lung histopathology. Infiltrates in lung tissue were observed in 45% of the lung sections of *Socs1^−/−^MGL^tg^* mice (Figure 7A) in three different founders. PAS staining was performed to identify mucus producing cells, revealing a higher number of PAS positive cells in the lungs of *Socs1^−/−^MGL^tg^* mice (Figure 7B). In addition, *Socs1^−/−^MGL^tg^* mice showed 19.4-fold increased serum IgE levels (Figure 7C). Since SOCS1 has been shown to be important for T helper cell differentiation (31, 49, 50), we analyzed whether *Socs1^−/−^MGL^tg^* mice have a T helper cell bias. Therefore, CD4^+^ T cells were isolated from lung homogenates and expression of transcription factors for T helper cell subsets was examined by qPCR (Figure 7D). *Socs1^−/−^MGL^tg^* mice showed a 4.2-fold increase of *Gata3*^+^ cells, suggesting a higher number of Th2 cells. Using an *in vitro* differentiation assay, naive CD4^+^ T cells from *Socs1^−/−^MGL^tg^* mice tend to express more Gata3 as compared to CD4^+^ T cells from *Socs1*^+/−^*MGL^tg^* mice, even under neutral conditions (T_0_, RPMI only) (Figure 7E). Increased mRNA expression of *IL-4, IL-5*, and *IL-13* in complete lung homogenates of *Socs1^−/−^MGL^tg^* mice compared to both *Socs*^+/−^*MGL^tg^* and *Socs*^+/−^ mice confirmed this Th2 bias (Figure 7F). Notably, we found one population of *Socs1^−/−^MGL^tg^* mice with a strong expression of *Gata3* and Th2 type cytokines in lung homogenates, and the second population showing a weaker Th2 bias, consistent with the fact that we could observe infiltrates in the lung only in 45% of the mice.

### Increased Airway Eosinophilia in *Socs1^−/−^MGL^tg^* Mice in an OVA Experimental Asthma Model

**Figure 8:**
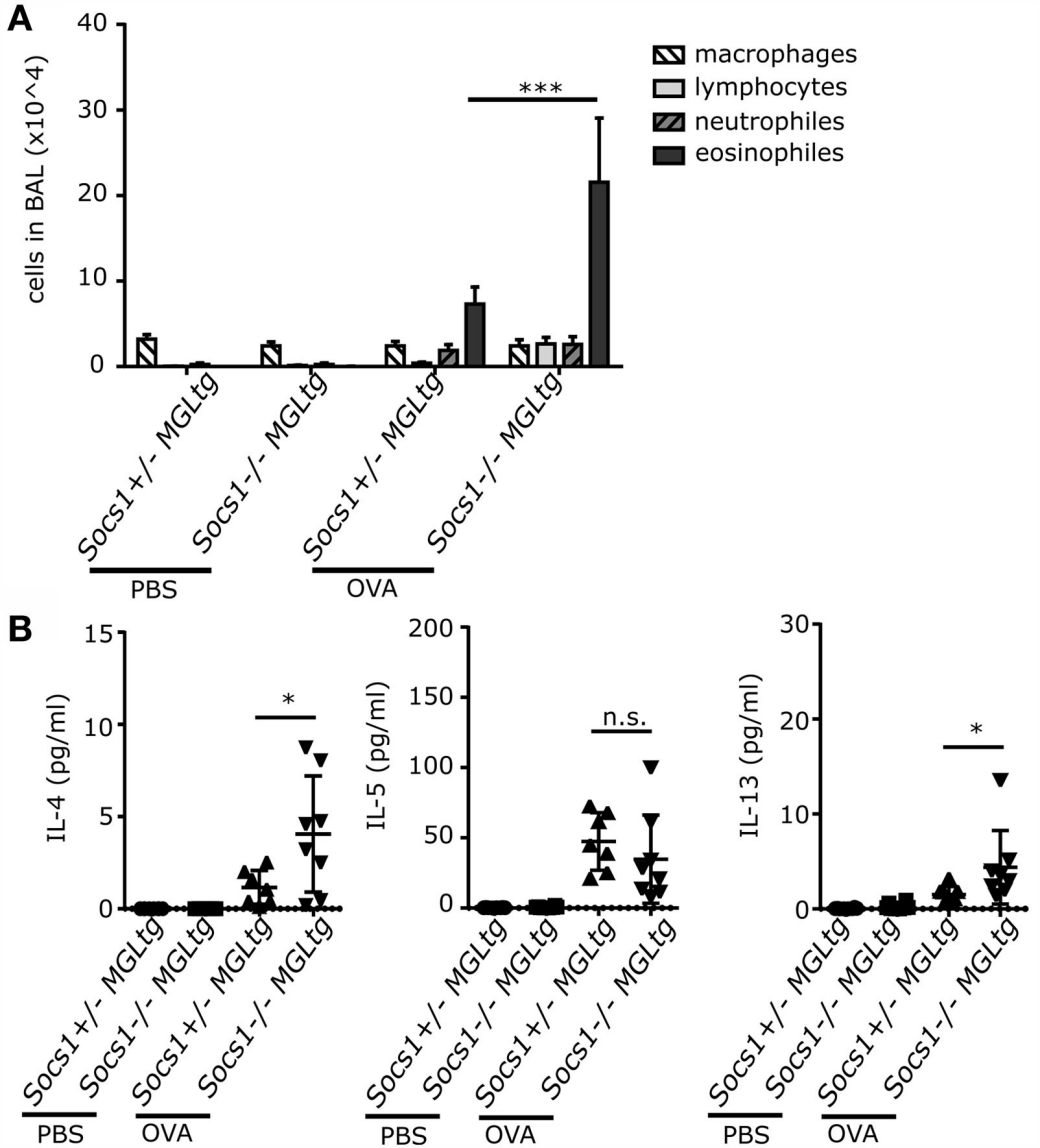
**Enhanced airway eosinophilia in *Socs1^−/−^MGL^tg^* mice upon OVA sensitization and challenge**. Mice were sensitized with OVA/Alum or PBS i.p. on days 1, 14, and 21, followed by a challenge with 1% OVA aerosol or PBS on days 26, 27, and 28. **(A)** Total numbers of leukocyte subpopulations in BAL fluids are represented. **(B)** Levels of IL-4, IL-5, and IL-13 in serum were measured by ELISA (*n* = 6–8, mean + SEM, two-way ANOVA including Bonferroni post-test).

To analyze if this Th2 bias is of physiological relevance, mice were challenged by inhaled antigen. *Socs1^−/−^MGL^tg^* mice were subjected to a well-established protocol for the induction of experimental asthma (41, 51). Upon OVA sensitization and OVA aerosol challenge, *Socs1^−/−^MGL^tg^* mice showed increased airway eosinophilia (21.5 × 10^4^ cells/ml in BALF) as compared to *Socs1*^+/−^*MGL^tg^* control mice (7.3 × 10^4^ cells/ml in BALF) (Figure 8A). In addition, IL-4 and IL-13 levels were higher in BAL fluid of *Socs1^−/−^MGL^tg^* mice upon OVA sensitization and challenge as compared to *Socs1*^+/−^*MGL^tg^* mice (Figure 8B). IL-5 levels upon challenge were induced, yet, showed no difference with respect to expression of the non-nuclear SOCS1. Similar effects were observed in *Socs1^−/−^MGL^tg^* mice upon intratracheal IL-13 instillation. IL-13-treated mice of all genotypes developed neutrophilia in the lung. *Socs1^−/−^MGL^tg^* mice additionally showed enhanced influx of eosinophils (however non-significant) and lymphocytes (Figure S4A in Supplementary Material). In addition, *Socs1^−/−^MGL^tg^* mice showed increased mRNA expression of IL-4, IL-5, and IL-13, which was even more pronounced upon IL-13 treatment. *Socs1* induction in all three genotypes upon IL-13 instillation was equal (Figure S4B in Supplementary Material).

### Disrupted Epithelial Integrity in *Socs1^−/−^MGL^tg^* Mice

**Figure 9:**
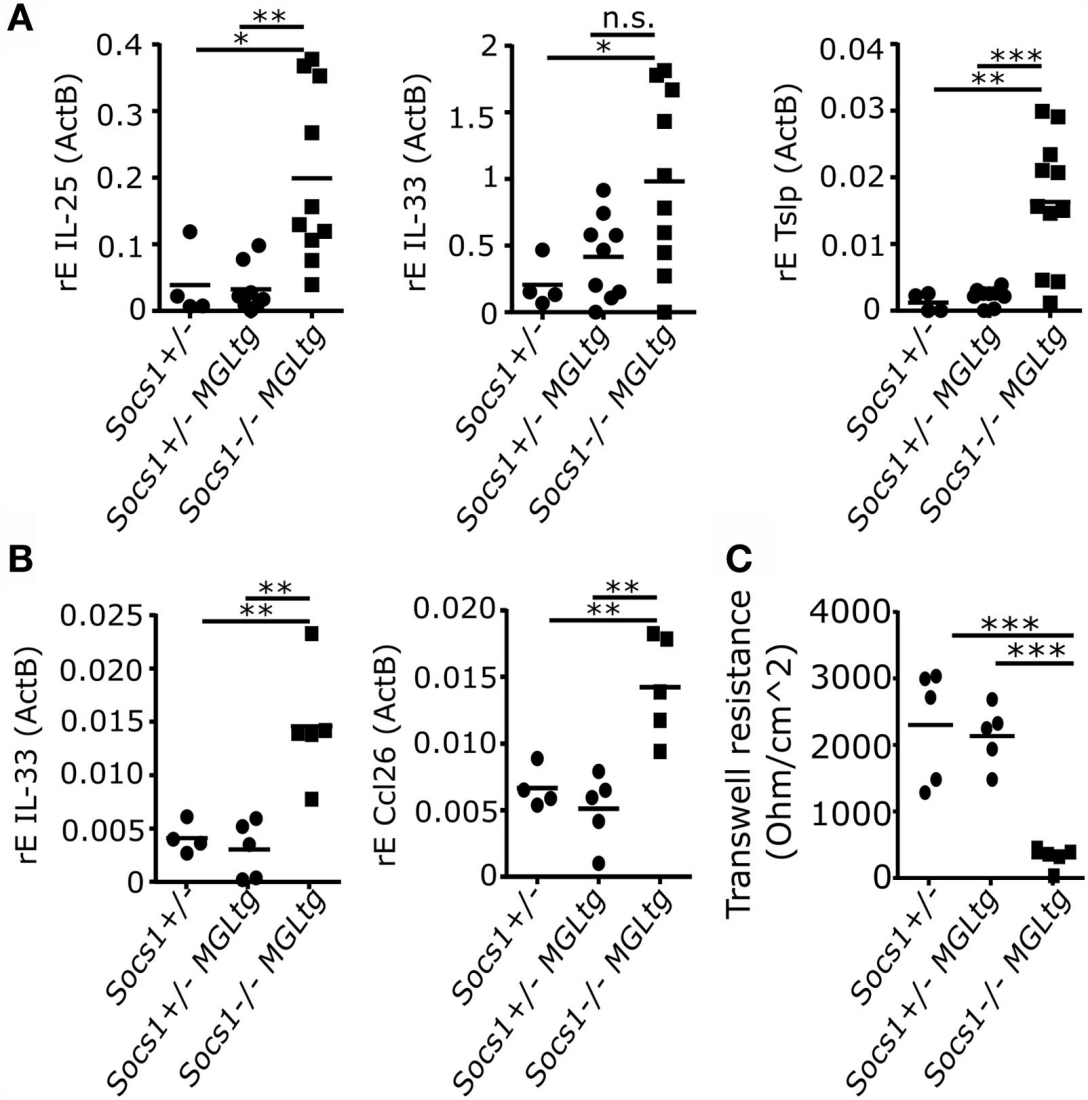
**Trachea epithelial cells from *Socs1^−/−^MGL^tg^* mice show alterations**. **(A)** Enhanced expression levels of *IL-25, IL-33*, and *Tslp* in total lung homogenates of both founder #53 and #29 (*n* = 7–11, mean + SD, one-way ANOVA including Bonferroni post-test). **(B)** Enhanced expression levels of *IL-33* and *Ccl26* in primary murine trachea epithelial cells (*n* = 5, mean + SD, one-way ANOVA including Bonferroni post-test). **(C)** Decreased transepithelial electrical resistance in trachea epithelial cells from *Socs1^−/−^MGL^tg^* mice as measured with the Millicell^®^ electrical resistance system (ERS) (*n* = 5, mean + SD, one-way ANOVA including Bonferroni post-test).

Since *Socs1^−/−^MGL^tg^* mice showed enhanced expression of IL-25, IL-33, and Tslp in lung homogenates (Figure 9A), we closer analyzed the airway epithelium. Therefore, tracheas were isolated and trachea epithelial cells were differentiated in an air–liquid interface (ALI) using transwells. Increased IL-33 expression could be verified in isolated trachea epithelial cells from *Socs1^−/−^MGL^tg^* mice. In addition, Ccl26 expression was examined since it is known for the recruitment of eosinophils (52). Indeed, trachea epithelial cells from *Socs1^−/−^MGL^tg^* mice expressed significantly more Ccl26 as compared to cells from *Socs1*^+/−^*MGL^tg^* mice. Interestingly, we found decreased TER in trachea epithelial cells from *Socs1^−/−^MGL^tg^* mice as compared to cells from *Socs1*^+/−^ and *Socs1*^+/−^*MGL^tg^* mice. This suggests disrupted epithelial integrity and might explain low-grade inflammation observed in the lungs of *Socs1^−/−^MGL^tg^* mice.

## Discussion

Suppressor of cytokine signaling 1 is a classical negative feedback regulator of cytoplasmic JAK/STAT signaling (4–6). However, it has been described that SOCS1 is also localized in the cell nucleus (33, 34), yet, the function of SOCS1 in the cell nucleus *in vivo* remains elusive. To study the role of nuclear SOCS1, we generated transgenic mice using a BAC containing a mutated *Socs1* (*Socs1ΔNLS*) that fails to translocate in the cell nucleus, which is expressed together with e*GFP* and *LuciferaseCBG99* (*MGL*). Using BACs to create transgenic mice is a commonly used approach (53–56), which allows manipulating genes embedded within their genetic regulatory environment. We aimed for similar gene regulation of *Socs1wt* and *Socs1ΔNLS* and therefore carefully controlled that the locus of BAC-vector integration produced similar transcript amounts of *Socs1wt* and *Socs1ΔNLS* (Figure 4). There were no detectable differences regarding expression and regulation of *Socs1wt* or *Socs1ΔNLS* mRNA between three different founders (Figure 2). Next, we investigated whether there is a gene dosage effect. We cannot fully exclude that increased localization of SOCS1ΔNLS to the cytoplasm contributes to the observed effects although we did not find any indication that this construct is more effective in inhibiting JAK/STAT signaling. In fact, data from *Socs1*^+/−^ and *Socs1*^+/−^*MGL^tg^* mice were very similar, thus arguing against any side effect due to increased cytoplasmic localization of SOCS1ΔNLS. In contrast to *Socs1^−/−^* mice, *Socs1*^+/−^ mice lacked pathological levels of IFNγ (24) and were found to be phenotypically normal (26). We confirm these data by showing that *Socs1*^+/−^ mice had normal survival (Figure 3) indicating that one allele of *Socs1* is sufficient for rescue of the severe knockout phenotype. Analyzing expression of IFNγ-dependent genes (Figure 5) and the lung phenotype (Figures 7 and 9), we found no difference between *Socs1*^+/−^ and *Socs1*^+/−^*MGL^tg^* mice, arguing against a gene dosage effect and for a localization-specific effect resulting in eosinophilic lung inflammation in *Socs1^−/−^MGL^tg^* mice. As reported previously, we found that SOCS1 is expressed at low levels and is relatively short-lived (57), but can be induced by IFNγ (49). The NLS of SOCS1 (RRMLGAPLRQRRVR, amino acid 159–173) resembles a bipartite NLS composed of two basic stretches. Lysine as a basic amino acid is important for the ubiquitin proteasome pathway, linking ubiquitin chains onto proteins to mark them for degradation *via* the proteasome (45, 46). Therefore, we addressed the question whether exchanging the NLS with the SOCS3 counterpart might alter protein half-life (Figures 4D,E). However, protein half-life was not altered upon exchanging the NLS corresponding part of SOCS1 with SOCS3 (SOCS1ΔNLS). In general, the results confirm previously described expression patterns of SOCS1, indicating that the transgene has integrated in a region accessible for transcriptional regulation and that using BAC transgenic mice is a valid approach to study the function of SOCS1 in the cell nucleus.

In order to show that *Socs1^−/−^MGL^tg^* mice indeed lack SOCS1 in the cell nucleus, we tried staining of SOCS1 on lung sections by immunohistochemistry. However, we could not find a specific antibody that was not staining sections from *Socs1^−/−^* mice. Therefore, we decided to apply a functional approach to verify non-nuclear expression of *Socs1MGL*. SOCS1 has been shown to induce proteasomal degradation of NFκB (36, 37) by interaction with p65 in the cell nucleus, thereby limiting induction of a subset of NFκB-dependent genes (38). Lack of nuclear SOCS1 leads to sustained activation of NFκB that could be confirmed using a transcription factor assay specifically for p65 (Figure S1 in Supplementary Material). *Socs1^−/−^MGL^tg^* mice indeed showed sustained IL-12p40 protein levels in CD11c^+^ cells of the lung and spleen (Figure 2F). We did not detect differences regarding TNFα protein levels (Figure S1 in Supplementary Material). Unlike *TNF*α that shows fast NFκB recruitment to a constitutively and immediately accessible promoter, *IL-12p40* is a gene that needs prolonged binding of NFκB to its promoter (44). Only a small subset of NFκB-dependent genes that is dependent on prolonged transcriptional activation is affected by sustained activation of p65 such as *IL-12p40*. Taken together, findings in *Socs1^−/−^MGL^tg^* mice are fully consistent with previously described *in vitro* data using non-nuclear SOCS1ΔNLS (38), suggesting that *Socs1^−/−^MGL^tg^* mice lack SOCS1 in the cell nucleus. In addition, we found a substantial number of differentially expressed genes annotated to TLR and TNF signaling, and NFκB-binding sites were overrepresented among those genes (Tables 1 and 2).

*Socs1^−/−^MGL^tg^* mice, expressing only non-nuclear mutant *Socs1* (*Socs1ΔNLS*), survive the early lethal phenotype of *Socs1^−/−^* mice (Figure 3; Figure S2 in Supplementary Material) that otherwise die within 3 weeks due to excessive immune signaling and multiorgan inflammation (24–26). The data show that SOCS1ΔNLS was sufficient to rescue lack of wild-type SOCS1. The disease in *Socs1* knockout mice mainly depends on hyperresponsiveness to IFNγ as it can be prevented in the neonatal period by the administration of anti-IFNγ antibodies or by using *Socs1^−/−^IFNγ^−/−^* mice (47, 48). Therefore, we hypothesized that canonical IFNγ signaling might still be efficiently regulated by Socs1ΔNLS. IFNγ binds to the IFNγ receptor complex, activates JAK1/2, and subsequently leads to tyrosine phosphorylation of STAT1 (pY-STAT1). pY-STAT1 dimers in turn translocate into the nucleus and activate transcription of “canonical” IFNγ-responsive genes (15, 16, 58). *Socs1^−/−^MGL^tg^* mice showed functional regulation of canonical IFNγ signaling, as shown by unaltered pY-STAT1 levels and whole-genome expression analysis in BMMs (Figures 5 and 6). In addition to canonical signaling, a number of studies have shown that pY-STAT1-independent pathways also exist (17–19). Besides their localization on the plasmamembrane, IFNAR1 and TYK2 have been shown to occur in the nucleus as well (21, 22). In addition, it has been shown that STATs translocate into the nucleus in a pY-independent manner, where they activate expression of only a subset of “non-canonical” IFNγ-induced genes (23). Indeed, a subset of non-canonical IFNγ target genes were differentially regulated comparing BMMs of *Socs1^−/−^MGL^tg^* and *Socs1*^+/−^*MGL^tg^* mice. Pathway annotation did not reveal IFNγ signaling to be differentially regulated, confirming that Socs1ΔNLS was still able to regulate cytoplasmic signaling pathways and that canonical IFNγ signaling was not altered in *Socs1^−/−^MGL^tg^* mice. We observed minor, but non-significant differences in *Icam-1* expression upon stimulation with IFNγ comparing BMMs of *Socs1*^+/−^*MGL^tg^* and *Socs1^−/−^MGL^tg^* mice (Figure 5). This is in line with literature showing that upregulation of ICAM-1 by IFNγ is inhibited by SOCS1 (59) with its inhibitory capacity depending on the functional NLS of SOCS1 (34).

We closely analyzed *Socs1^−/−^MGL^tg^* mice for disease symptoms and found reduced body weight and spontaneous development of low-grade inflammation in the lung (Figure 7). Expression of SOCS1 in the lung has been reported for alveolar macrophages (60), bronchial epithelial cells (61), and eosinophils (62). Mice fully deficient for SOCS1 show extensive hematopoietic infiltration in the lung (26), arguing that SOCS1 is involved in immune regulation in the lung. Increased serum IgE levels in *Socs1^−/−^MGL^tg^* mice suggest an allergic airway disease. To analyze whether the Th2 bias observed in *Socs1^−/−^MGL^tg^* mice is of physiological relevance, mice were challenged by either inhaled OVA or IL-13. Both, upon OVA sensitization and challenge as well as IL-13 instillation, *Socs1^−/−^MGL^tg^* mice showed increased airway eosinophilia (Figure 8; Figure S4 in Supplementary Material). This is in line with previous data showing that serum IgE levels and infiltrating eosinophils were considerably increased in the lungs of OVA-treated *Socs1^−/−^IFNγ^−/−^* mice (63). So far, it is unclear how the lack of nuclear SOCS1 leads to airway eosinophilia. One hypothesis is that SOCS1 is crucial to maintain epithelial cell barrier function (Figure S5 in Supplementary Material). Sustained NFκB signaling might lead to an activation of the epithelium. We observed increased expression of the epithelial cell-derived cytokine *IL-33* in primary murine trachea epithelial cells of *Socs1^−/−^MGL^tg^* mice (Figure 9). Since it has been shown previously (64) that IL-33 has an impact on epithelial integrity, higher IL-33 levels in *Socs1^−/−^MGL^tg^* mice might result in epithelial barrier disruption. Enhanced barrier permeability in turn might facilitate other immune cells such as DCs in initiate host defense mechanisms resulting in inflammation (65). Triggering of pattern recognition receptors on epithelial cells has been reported to release of IL-33 leading to an activation of DCs (66–68). Recently, it has been shown that IL-33 is constitutively expressed in the cell nucleus in epithelial cells (69) where direct interaction between SOCS1 and IL-33 might be possible. Furthermore, IL-13 has been shown to downregulate junctional components including E-cadherin in bronchiolar epithelial cells leading to disruptive effects on airway epithelial barrier function (70) and explaining why we see stronger eosinophilia upon IL-13 treatment. Indeed, higher SOCS1 expression has been shown to inhibit IL13 induced CCL26 expression in epithelial cells *in vitro* whereas reduced SOCS1 expression was correlated with enhanced airway eosinophilia (71). Epithelial cells of *Socs1^−/−^MGL^tg^* mice produce more CCL26 which in turn attracts eosinophils resulting in airway eosinophilia (Figure S5 in Supplementary Material).

The second hypothesis is that hematopoietic cells are the key players involved in nuclear SOCS1 induced airway eosinophilia. Lee et al. showed that serum IgE levels and infiltrating eosinophils were considerably increased in the lungs of OVA-treated *Socs1^−/−^IFNγ^−/−^* mice (63). They suggest that regulation of SOCS1 mainly affects hematopoietic cells, not epithelial cells. McCormick et al. showed that reduced expression of SOCS1 has been shown to prolong IL-4-induced IRS-2 tyrosine phosphorylation and enhanced M2 differentiation (72). IRS-2 also plays a major role in allergic lung inflammation and remodeling (73). In addition, SOCS1 is important in helper T cell differentiation (5, 6, 49): it is rapidly induced in response to many cytokines, including IFNγ and IL-4 and it is an important negative feedback inhibitor of both signaling pathways. When *Socs1^−/−^* mice are crossed with either an *IFNγ^−/−^* or *STAT6^−/−^* mice, survival is prolonged (47, 50), indicating that SOCS1 regulates both IFNγ-driven Th1 and IL-4-driven Th2 responses. Supporting this finding, CD4^+^ T cells from *Socs1^−/−^* mice spontaneously differentiate into Th1 and Th2 cells, thereby producing IFNγ and IL-4, respectively (31, 50). It has previously been shown *in vitro* that SOCS1 is a negative regulator of Th2-dependent pathways, achieved by inhibition of pSTAT6 (74). In line with this, *Socs1^−/−^MGL^tg^* mice showed enhanced percentage of *Gata3*^+^ CD4^+^ cells and increased expression of *IL-4, IL-5*, and *IL-13*, suggesting that nuclear SOCS1 plays a role in T cell differentiation. Even under neutral conditions, CD4^+^ T cells of *Socs1^−/−^MGL^tg^* mice tend to differentiate into Gata3^+^ cells, arguing for a T cell intrinsic effect of nuclear SOCS1. Increased Th2 cytokines in *Socs1^−/−^MGL^tg^* mice could in turn act on the epithelial cells. Further clarification will require generating bone marrow chimeras to differentiate between contributions of nuclear SOCS1 in cells sensitive for radiation (hematopoietic cells) or radiation-resistant cells (such as epithelial cells).

There have been several publications linking SOCS1 expression with allergic diseases such as asthma (61, 71, 74, 75). Gielen et al. (61) observed that nuclear SOCS1 suppressed rhinovirus induction of interferons, which is discussed to be associated with increased susceptibility to virus exacerbation in severe asthma. Since induction of interferons occurs *via* pattern recognition receptors that are linked to NFkB signaling, inhibition of NFkB signaling by nuclear SOCS1 might be a possible mechanism for the control of immunity in the lung. *Socs1* gene expression was significantly lower expressed in the airways of severe asthmatics compared with mild/moderate asthmatics, and was inversely associated with airway eosinophilia (71, 74), suggesting that the absence of SOCS1 leads to Th2 bias. Using *Socs1^−/−^MGL^tg^* mice, we have shown for the first time, that not only the presence of SOCS1 but also the localization is crucial for effective regulation of Th2 responses. A study assessing functional variants of *Socs1* within a population of adult Japanese asthma patients found a significant association between the *Socs1* promoter polymorphism (−1478CA < del) and adult asthma. It is suggested that promoter polymorphism leads to increased SOCS1 and inhibition of interferons, leading to higher susceptibility to virus-induced asthma exacerbations (75). Another study showed that expression of nuclear SOCS1 is increased in atopic asthmatic patients (61), which was associated with suppression of rhinovirus-induced interferons. The findings allow the conclusion that SOCS1 in the cell nucleus plays an important role in the regulation of local immunity in the lung that needs to be further investigated.

Taken together, *Socs1^−/−^MGL^tg^* mice showed functional regulation of canonical IFNγ signaling, but differential regulation of a subset of IFNγ-dependent genes, possibly due to alteration in NFκB signaling pathway. *Socs1^−/−^MGL^tg^* mice spontaneously developed low-grade airway inflammation and had increased serum IgE levels and Th2 cytokines in the lung. Upon OVA sensitization and OVA aerosol challenge as well as IL-13 instillation, *Socs1^−/−^MGL^tg^* mice reacted with augmented influx of eosinophils, arguing for an immune regulatory function of nuclear SOCS1 in the lung. We present a valuable tool to study the nuclear function of SOCS1 *in vivo* that allows investigating local immune regulation in the lung by nuclear SOCS1.

## Author Contributions

BA, MWg, and AD designed the work and interpreted data. JZ, MWt, ST, FL, LL, ZO, and CV acquired and analyzed experimental data. BA, GK, PW, and AD generated the BAC transgenic mouse. SB analyzed and interpreted data. JZ, MWg, MWt, and AD wrote the manuscript.

## Conflict of Interest Statement

The authors declare that the research was conducted in the absence of any commercial or financial relationships that could be construed as a potential conflict of interest.
