# Supplementary material for: Nuclear Localization of Suppressor of Cytokine Signaling-1 Regulates Local Immunity in the Lung
Source: Front Immunol. 2016 Nov 18;7:514. doi: 10.3389/fimmu.2016.00514 (PMC5114302; doi:10.3389/fimmu.2016.00514)
Supplement: Supplementary file 1 [file Data_Sheet_1.pdf]

Supplementary Figures

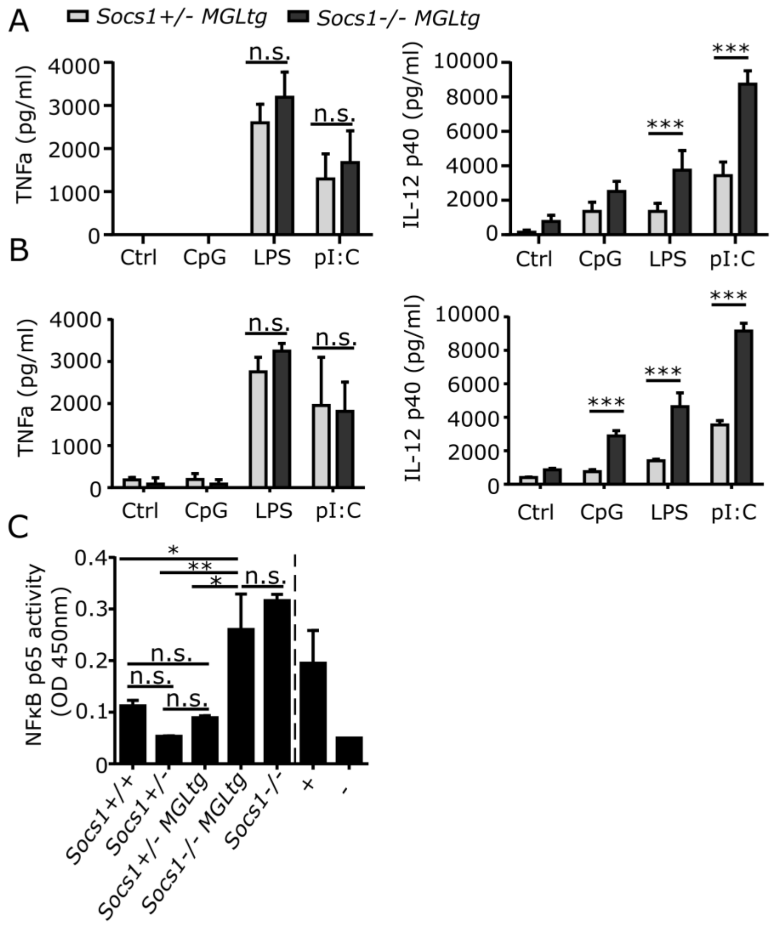

Fig. S1 Sustained NFκB signaling in *Socs1*$^{-/-}$*MGL*^{tg} mice. CD11c^{+} cells were isolated from (A) lung and (B) spleen homogenates and stimulated with CpG (1 µM), LPS (100 ng/ml) and pI:C (10 µg/ml) for 24 h. TNFα and IL-12p40 protein levels were measured by ELISA (n= 3- 5, mean +SD, Two-way ANOVA). (Fig. S1A right panel is also shown in Fig. 2F). (C) NFκB p65 activity was examined using the TransAM® DNA binding ELISA to specifically analyze p65 activity in lung homogenates of *Socs1*$^{+/+}$, *Socs1*$^{+/-}$, *Socs1*$^{+/-}$*MGL*^{tg}, *Socs1*$^{-/-}$*MGL*^{tg} and *Socs1*$^{-/-}$*MGL*^{tg} mice. Raji nuclear extract was used as a positive control (+) and a wildtype oligonucleotide was used as a competitor (-) (n = 2, mean +SD, One-way ANOVA including Bonferroni post test).

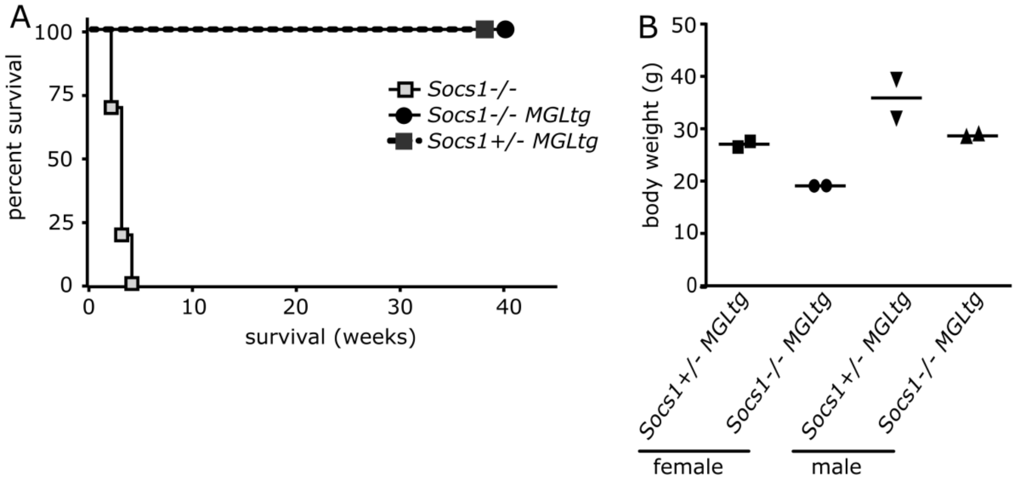

Fig. S2 Long-term survival. (A) Survival was recorded for n= 28 *Socs1*$^{-/-}$ and n=4 *Socs1*$^{+/-}$*MGL*^{tg} and n=4 *Socs1*$^{-/-}$*MGL*^{tg} mice up to 38 weeks. (B) Body weight was recorded in the same cohort (n = 2 per genotype and gender, mean +SD, Wilcoxon matched pairs test).

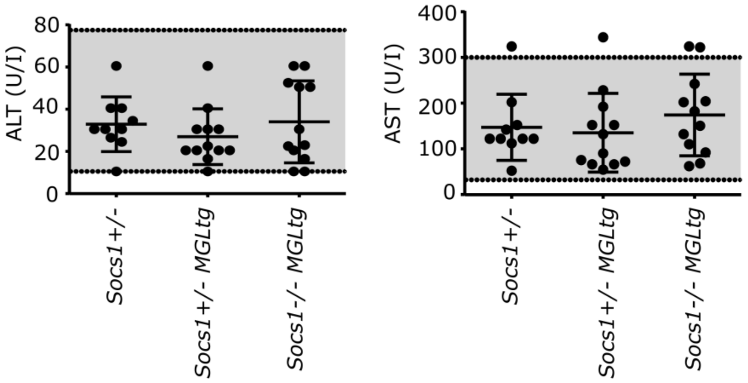

Fig. S3 Serum transaminase levels at an age of 8 - 12 weeks. Serum AST and ALT levels were determined in n= 10 *Socs1*$^{+/-}$ and n= 12 *Socs1*$^{+/-}$*MGL*^{tg} and *Socs1*$^{-/-}$*MGL*^{tg} mice.

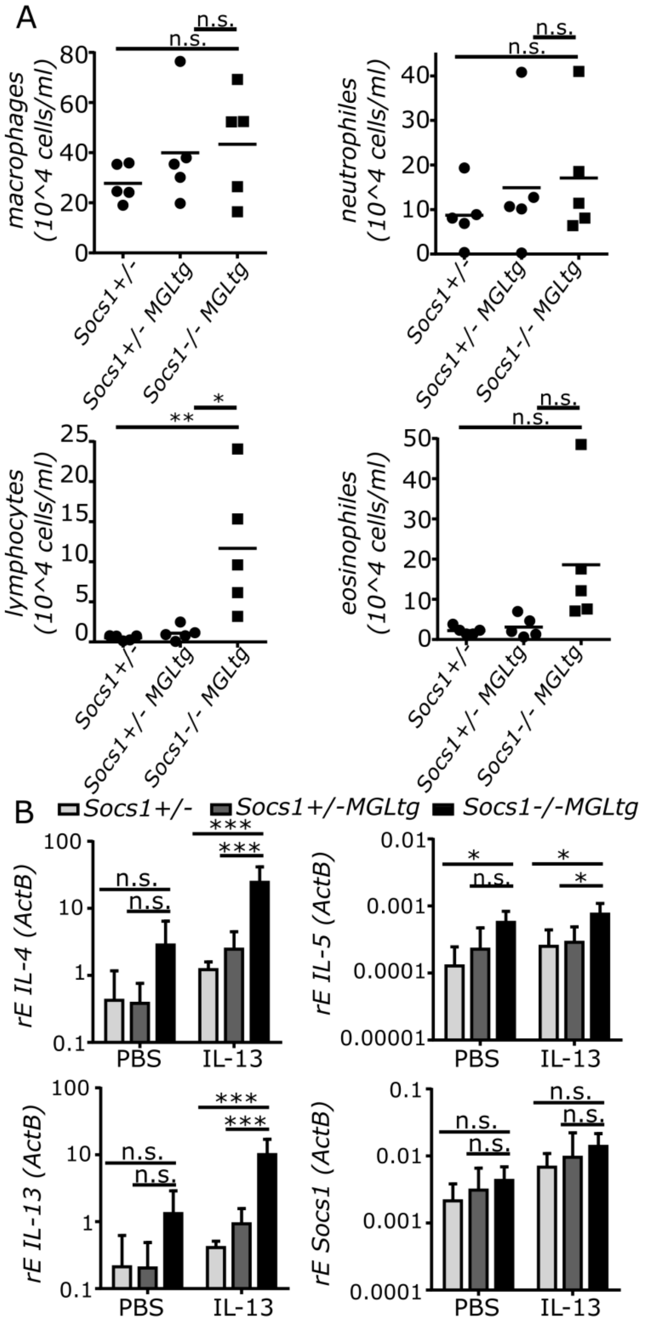

Fig. S4 Enhanced airway eosinophilia in *Socs1*$^{-/-}$*MGL*^{tg} mice upon IL-13 instillation. Mice were anesthetized with isofluorane and 20 µl of PBS with or without 5 µg IL-13 were applied intratracheally on days 1, 2 and 3. Analysis was performed on day 4 (A) Total numbers of leukocyte subpopulations in BAL fluids are represented (n = 5, mean +SD, One-way ANOVA including Bonferroni post test). (B) Levels of *IL-4*, *IL-5*, *IL-13* and *Socs1* were measured by qPCR (n = 5, mean +SD, Two-way ANOVA including Bonferroni post test).

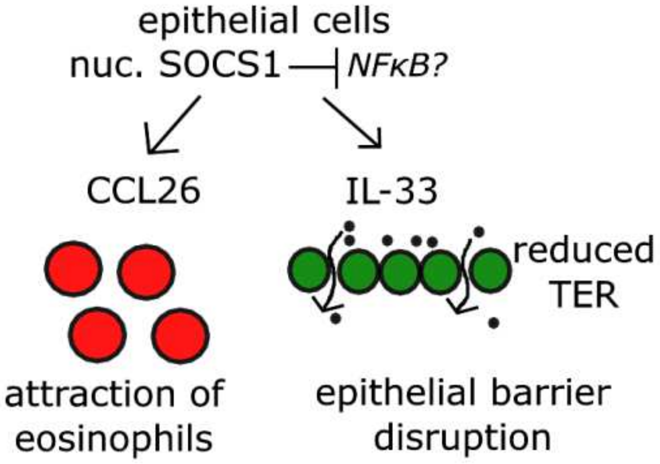

Fig. S5 Scheme on how nuclear SOCS1 might influence airway eosinophilia. Sustained NFκB signaling in *Socs1*$^{-/-}$*MGL*^{tg} mice due to missing nuclear SOCS1 might lead to an activation of the epithelium. Increased production of Ccl26 in turn attracts eosinophils to the airways. In addition, epithelial cells of *Socs*$^{-/-}$*MGL*^{tg} mice produce more IL-33, which has an impact on epithelial integrity. Enhanced barrier permeability might facilitate other immune cells to initiate host defense mechanisms leading to low-grade inflammation. How SOCS1 acts on epithelial cells and whether this is an NFκB dependent process is currently unknown.

Supplementary Tables

Table S1 Primer sequences.

Primer	Sequence (fw) (5'-3')	Sequence (rv) (3'-5')
ActB	CCCTGTGCTTGGCTTCACCGA	ACAGTGTGGGTGACCCCGTTC
iNOS	CAGCTGGGCTGTACAAACCTT	CATTGGAAGTGAAGCGTTTCG
Irf9	GCCGAGTGGTGGGTAAGAC	GCAAAGGCGCTGAACAAAGAG
Icam-1	GGCATTGTTCTCTAATGTCTCCG	TGTCGAGCTTTGGGATGGTAC
IL-4	GGTCTCAACCCCCAGCTAGT	GCCGATGATCTCTCTCAAGTGAT
IL-5	CTCTGTTGACAAGACCTG	TCTTCAGTATGTCTAGCCCCTG
IL-13	CCTGGCTCTTGCTTGCCTT	GGTCTTGTGTGATGTTGCTCA
IL-25	ACAGGGACTTGAATCGGGTC	TGGTAAAGTGGGACGGAGTTG
IL-33	TCCTTGCTTGGCAGTATCCA	TGCTCAATGTGTCAACAGACG
Tslp	ACGGATGGGGCTAACTTACAA	AGTCCTCGATTTGCTCGAACT
Ccl26	TTCTTCGATTTGGGTCTCCTTG	GTGCAGCTCTTGTCGGTGAA
Tbet	AGCAAGGACGGCGAATGTT	GGGTGGACATATAAGCGGTTC
Gata3	CTCGGCCATTCGTACATGGAA	GGATACCTCTGCACCGTAGC
Rorc	GACCCACACCTCACAAATTGA	AGTAGGCCACATTACACTGCT
Foxp3	CCCATCCCCAGGAGTCTTG	ACCATGACTAGGGGCACTGTA
***Socs1ΔNLS* (P1 & P3)**	CACCTTCTTGGTGCGCG	GAGGAGAGAGGTCGGCTCAGTAC
***Total Socs1* (P1 & P2)**	CACCTTCTTGGTGCGCG	CCCCCAACATGCGGCGCG
***Socs1 wt* (probe)**	FAM-ATGTTGGGGGCCCCGCTGCG-BHQ_2	

***Socs1 wt* (Genotyping)**	GCATCCCTCTTAACCCGGTAC	AAATGAAGCCAGAGACCCTCC
***Socs1 MGL* (Genotyping)**	*TAAACGGCCACAAGTTCAGC*	*TTCATGTGGTCGGGGTAGC*
***Socs1 KO* (Genotyping)**	TCCAGCTGGCCCCTCGAGTAGGATG	ATTCGCCATTCAGGCTGCGCAACTGTT
***β2M* (Genotyping)**	CACCGGAGAATGGGAAGCCGAA	TCCACACAGATGGAGCGTCCAG

Table S2 Histopathological Analysis**.** Organs were cut at 3 µm, H&E stained and histopathologically evaluated. n= 24 *Socs1*$^{-/-}$ *MGL*^{tg} and 12 *Socs1*$^{+/-}$ *MGL*^{tg} mice. * Fisher's exact test

**Infiltrating cells in**	***Socs1***$^{-/-}$ ***MGL tg* (%)**	***Socs1***$^{+/-}$ ***MGLtg* (%)**	**p-value ***	**risk-factor**
**Lung**	45	8	0.03	5.5
**Liver**	54	16	0.04	3.3
**Esophagus**	33	8	n.s.	4
**Small intestine**	29	0	n.s.	3
**Heart**	29	0	n.s.	2
**Stomach**	25	8	n.s.	/
**Spleen**	16	8	n.s.	/
**Skin**	12	0	n.s.	/
**Kidney**	8	0	n.s.	/
**Muscle**	4	0	n.s.	/
**Brain**	4	0	n.s.	/
**Colon**	4	0	n.s.	/
**Pancreas**	0	0	n.s.	/
**Parotis**	0	0	n.s.	/
